# Advances in Electrochemical Biosensors Based on Nanomaterials for Protein Biomarker Detection in Saliva

**DOI:** 10.1002/advs.202205429

**Published:** 2022-12-30

**Authors:** Tao Dong, Nuno Miguel Matos Pires, Zhaochu Yang, Zhuangde Jiang

**Affiliations:** ^1^ Chongqing Key Laboratory of Micro‐Nano Systems and Intelligent Transduction Collaborative Innovation Center on Micro‐Nano Transduction and Intelligent Eco‐Internet of Things Chongqing Key Laboratory of Colleges and Universities on Micro‐Nano Systems Technology and Smart Transducing National Research Base of Intelligent Manufacturing Service Chongqing Technology and Business University Nan'an District Chongqing 400067 China; ^2^ Department of Microsystems‐ IMS Faculty of Technology Natural Sciences and Maritime Sciences University of South‐Eastern Norway‐USN P.O. Box 235 Kongsberg 3603 Norway; ^3^ State Key Laboratory for Manufacturing Systems Engineering International Joint Laboratory for Micro/Nano Manufacturing and Measurement Technology Xi'an Jiaotong University Xi'an 710049 China

**Keywords:** biosensors, electrochemical sensor materials, nanomaterials, point‐of‐care systems, protein biomarkers

## Abstract

The focus on precise medicine enhances the need for timely diagnosis and frequent monitoring of chronic diseases. Moreover, the recent pandemic of severe acute respiratory syndrome coronavirus 2 poses a great demand for rapid detection and surveillance of viral infections. The detection of protein biomarkers and antigens in the saliva allows rapid identification of diseases or disease changes in scenarios where and when the test response at the point of care is mandated. While traditional methods of protein testing fail to provide the desired fast results, electrochemical biosensors based on nanomaterials hold perfect characteristics for the detection of biomarkers in point‐of‐care settings. The recent advances in electrochemical sensors for salivary protein detection are critically reviewed in this work, with emphasis on the role of nanomaterials to boost the biosensor analytical performance and increase the reliability of the test in human saliva samples. Furthermore, this work identifies the critical factors for further modernization of the nanomaterial‐based electrochemical sensors, envisaging the development and implementation of next‐generation sample‐in‐answer‐out systems.

## Graphical abstract

This article reviews the recent developments of electrochemical sensors for the detection of disease‐signaling protein biomarkers in saliva bio‐samples. The role of nanomaterials in synergy with bio‐recognition probes and electrochemical sensing techniques for boosting the analytical performance of resultant sensors is described and discussed. Perspectives and views in this field are also given.

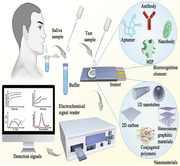

## 1 Introduction

Proteins are a class of macromolecules presented on the surface of infectious viruses and bacteria and are involved in key biological processes regulating the status of diseases. Differently expressed proteins can be the target of drugs and are often used as biomarkers for detection and diagnosis.^[^ ^1^, ^2^ ^]^ Hence, the early identification and quantification of proteins in body fluids are vital to the control of infectious and chronic diseases.

Body fluids including blood, urine, interstitial fluid, and saliva are commonly targeted in the measurement of protein biomarkers.^[^ ^3^ ^]^ Saliva in particular is preferable for testing in point‐of‐care (POC) settings where rapid diagnosis is demanded.^[^ ^4^ ^]^ Saliva can be easily and repetitively collected in sufficient volumes for detection, involving a low risk of infection and the procedure is quite patient‐compliant. Therefore, a powerful method of rapid disease tracing and detection can be developed by targeting saliva as the diagnostic fluid and analyzing it with a sensor with simple operation and high analytical performance.

The global pandemic of severe acute respiratory syndrome coronavirus 2 (SARS‐CoV‐2) has stimulated the rapid development of saliva analysis tools. During the last 2 years, a great number of saliva‐based sensor technologies for SARS‐CoV‐2‐related proteins, namely viral antigens or SARS‐CoV‐2 antibodies, have been reported.^[^ ^5^, ^6^, ^7^, ^8^ ^]^ The positive test for SARS‐CoV‐2‐related proteins rapidly identifies the infected individuals in situations where and when the RT‐PCR test is not available. The clinical value of saliva‐based protein sensors is extended to other infectious disease diagnostics (i.e., rapid tests for malaria),^[^ ^9^ ^]^ personalized medicine (i.e., routine tests for chronic lung disease or heart disease),^[^ ^10^, ^11^ ^]^ and other medical conditions affecting millions of people (i.e., on‐site tests for physiological stress detection, early‐stage detection of cancer, etc.).^[^ ^12^, ^13^, ^14^ ^]^

Protein detection methods applied to saliva have been benefiting from the standardization of specimen collection through the use of modern non‐invasive devices.^[^ ^15^ ^]^ However, several challenges persist with saliva‐based protein detection. In the first place, the protein biomarkers are present at significantly low concentrations in saliva, which poses a challenge to the resolution and detection limits of current analysis tools. Second, the target proteins may vary in concentration by several orders of magnitude, adding to the difficulty in terms of detection range. Finally, the specific detection of salivary protein can be hampered by interfering molecules and ions present in saliva.^[^ ^16^ ^]^ The exploitation of nanomaterials in conjugation with advanced sensor architectures may constitute a pathway to mitigate those challenges.

### 1.1 Overview of Salivary Protein Detection

Analyses of protein biomarkers in saliva are typically conducted by the enzyme‐linked immunosorbent assay (ELISA).^[^ ^17^ ^]^ The common procedure involves the immobilization of the protein on the ELISA microtiter plate either by direct adsorption or by indirect immobilization using a capture antibody pre‐coated on the plate. The detection is executed by loading an enzyme‐labeled antibody whose interaction with a chemical substrate produces a colorimetric or a luminescent signal. Western blotting is another immunological‐based method more commonly used in confirmation studies of candidate salivary biomarkers.^[^ ^18^ ^]^ Gel electrophoresis is employed in this technique with the separated proteins visualized on PVDF membranes using specific antibodies coupled with either radio‐conjugates or enzyme labels.

Screening of the saliva proteome with high throughput has been performed by liquid chromatography/tandem mass spectrometry (LC‐MS/MS). Hsiao et al. for instance have exploited multiplexed LC‐MS/MS assays to screen hundreds of peptides representing sets of protein biomarkers.^[^ ^19^ ^]^ The study has characterized variabilities in 90 proteins among samples collected from the same individual and samples collected from different individuals. For the analysis of up to a few hundred proteins, the use of gel electrophoresis coupled to MS is a common procedure; however, the need for analyzing low‐abundance salivary proteins (as in the case of interleukins for instance) calls for advanced MS techniques such as matrix‐assisted laser desorption/ionization time‐of‐flight (MALDI‐TOF/TOF) and linear ion trap. These techniques would ensure high resolution for salivary protein identification.^[^ ^20^ ^]^ By utilizing an assay similar to that reported by Hsiao et al., Kipping et al. have screened surrogate peptides derived from SARS‐CoV‐2 nucleocapsid protein.^[^ ^21^ ^]^ Despite the broad versatility and rapid analysis time (within a few minutes), the technique requires intensive protocols for sample pre‐treatment and target labeling before the analysis.

Raman spectroscopy is an alternative tool for protein profiling with much simpler sample preparation compared with LC‐MS/MS. The technique creates fingerprints of proteins based on the phenomenon of Raman scattering which originates from a frequency shift in the radiation of a laser upon to interaction of light with proteins. Typically, the Raman signal needs to be enhanced by the use of metallic nanostructures exploiting chemical enhancement or amplification via surface plasmon resonance. Surface‐enhanced Raman spectroscopy (SERS) has been evaluated for profiling various salivary cytokines as potential biomarkers of asthma.^[^ ^22^ ^]^ Gold (Au) nanorods were employed as signal enhancers, and the method has shown good accuracy for the early identification of bronchial inflammation in asthmatic patients. Silver nanoparticles were used in another study revealing the potential of SERS to decipher the diagnosis of lung cancer patients compared to the control group by analyzing salivary protein signatures.^[^ ^23^ ^]^ Molecular “barcodes” can be constructed using SERS and the combination with advanced machine learning techniques for further signal processing enhances the detection accuracy.^[^ ^24^ ^]^ Alterations in immunoglobulin and other proteins were identified by attenuated total reflection‐Fourier transform infrared spectroscopy which exhibited a significant power of discrimination between SARS‐CoV‐2 infected patients and healthy individuals.^[^ ^25^ ^]^ The technique can also generate diagnostic fingerprints from saliva samples in combination with multivariate analysis.

The confinement of the immunological methods, LC‐MS/MS, and spectroscopic‐based techniques to the clinical chemistry laboratory hinders, despite their reliability, the use of these techniques for rapid diagnosis of diseases or rapid feedback on results of disease treatment. The advantage of saliva‐based diagnostics is centered on the sample's easy accessibility, which is ideal for POC settings. The use of complex instrumentation and laborious operations of the assay prevent the test from providing the detection result immediately. The problem has motivated the development of protein biosensors^[^ ^26^ ^]^ which can provide detection results in minutes and are amenable to analyzing the biofluid sample in settings outside a clinical laboratory. Various biosensors have been studied for detecting protein biomarkers in saliva, including electronic sensors,^[^ ^27^ ^]^ electrochemical sensors,^[^ ^17^ ^]^ fluorescent sensors,^[^ ^28^ ^]^ interferometer sensors,^[^ ^6^ ^]^ plasmonic sensors,^[^ ^29^ ^]^ absorbance sensors,^[^ ^30^ ^]^ and quartz crystal microbalance sensors.^[^ ^31^ ^]^ However, many of the reported technologies do not exhibit sufficient analytical performance and easy operation in the field. Satisfying the growing demand for saliva‐based biosensors with the characteristics of low cost, ultra‐high sensitivity, and fast test cycles from sampling to analyte detection remains an important challenge nowadays.^[^ ^32^ ^]^ The electrochemical sensor may offer the best compromise between low cost and high analytical performance among the existing sensor platforms. The electrochemical sensor is acknowledged to be sensitive, fast, with low detection limits, easily integrated, and amenable to miniaturization at reasonable costs.^[^ ^16^, ^33^, ^34^, ^35^, ^36^ ^]^ These characteristics make the electrochemical assay suitable for perfect POC devices. Nevertheless, when challenged with the saliva sample, the sensor shall exhibit superior selectivity to the target due to the complex composition of the sample, as well as superior sensitivity and detection limit as the salivary protein biomarkers are commonly present in the sample at low concentration compared with blood for instance. Nanomaterials and related composites are often selected as the technology solution to enhance the analytical merits of the electrochemical sensor while retaining its costs and miniaturization advantages. Various classes of nanomaterials have been used to modify the electrode surfaces, including Au nanoparticles,^[^ ^37^ ^]^ carbon nanotubes,^[^ ^38^ ^]^ magnetic nanoparticles,^[^ ^39^ ^]^ exfoliated graphene,^[^ ^40^ ^]^ reduced graphene oxide,^[^ ^41^ ^]^ metal oxide nanoparticles,^[^ ^42^ ^]^ metal‐based thin‐films,^[^ ^43^ ^]^ and organic‐based thin‐films.^[^ ^44^ ^]^

This review intends to present the latest advances in electrochemical sensing for protein assays in saliva, focusing on the routes to be explored for the deployment of nanomaterial‐based electrochemical sensors in this field. Current and future sensor platforms incorporating nanomaterials or their associated composites are outlined in this paper.

### 1.2 Disease‐Marker Proteins in Saliva

Salivary proteins have been associated with various diseases, including localized and systemic diseases. **Table** **1** lists examples of salivary proteins targeted as biomarkers of diseases.

**Table 1:** Summary of clinically relevant protein biomarkers in saliva

Protein biomarker	Physiological range^a)^	Disease	Ref.
Interleukin‐1*β*	161.51^c)^–1312.75^d)^ (pg mL^−1^)	Periodontitis	[45]
Interleukin‐6	10^c)^–>30^d)^ (pg mL^−1^)	Periodontitis	[46]
	0.6^c)^–43.6^d)^ (pg mL^−1^)	Oral squamous cell carcinoma	[47]
Interleukin‐8	210.10^c)^–1718.61^d)^ (pg mL^−1^)	Oral squamous cell carcinoma	[48]
Interleukin‐2	2.07^c)^–3.06^d)^ (U mL^−1^)	Mucositis in individuals with acute lymphoblastic leukemia	[49]
Vascular endothelial growth factor—VEGF	280^c)^–4321^d)^ (pg mL^−1^)	Oropharyngeal cancer	[50]
Tumor necrosis alpha—TNF‐*α*	8.60^c)^–27.75^d)^ (pg mL^−1^)	Oral squamous cell carcinoma	[51]
	2.15^c)^–12.92^d)^ (pg mL^−1^)	Periodontitis	[52]
Human epidermal growth factor receptor 2—HER2	9.93^e)^–146.70^f)^ (pg mL^−1^)	Breast cancer	[53]
Triosephosphate isomerase—TPI1	>800^c)^–<400^d)^ (U mL^−1^)	Gastric cancer	[54]
Matrix metalloproteinase‐8—MMP‐8	190.91^c)^–348.26^d)^ (ng mL^−1^)	Periodontitis	[55]
Matrix metalloproteinase‐9—MMP‐9	145.87^c)^–231.02^d)^ (ng mL^−1^)	Sjögren´s syndrome	[56]
C‐reactive protein	<1^c)^–>80^d)^ (ng mL^−1^)	Cardiovascular disease	[57]
Secretory immunoglobulin A—sIgA	72.83^c)^–103.11^d)^ (mg L^−1^)	Human immunodeficiency virus infections	[58]
Immunoglobulin G	12.4^c)^–27.0^d)^ (mg per mg of total protein concentration)	Human papillomavirus infections	[59]
Cardiac troponin T	8.9^c)^–45.8^d)^ (pg mL^−1^)	Myocardial infarction	[60]
Galectin‐3	282.0^c)^–601.3^d)^ (ng mL^−1^) ^b)^	Heart failure	[61]
N‐terminal proB‐type natriuretic peptide—NT‐proBNP	<16^c)^–76.8^d)^ (pg mL^−1^) ^b)^	Heart failure	[62]
*α*‐amylase	<50–100^c)^–>150^d)^ (U mL^−1^)	Stress	[63]
Procalcinotin	0.09^c)^–0.50^d)^ (ng mL^−1^) ^b)^	Lung inflammation	[64]
Beta amyloid 42—A*β*42	21.1^c)^–51.7^d)^ (pg mL^−1^)	Alzheimer's disease	[65]
DJ‐1	<4^e)^–>5^f)^ (ng mL^−1^)	Parkinson's disease	[66]
SARS‐CoV‐2 nucleocapsid protein (N‐protein)	<1^c)^–100^d)^ (pg mL^−1^)	SARS‐CoV‐2 infections	[67]
SARS‐CoV‐2 IgG	7.0^c)^–25.5^d)^ (µg mL^−1^)	SARS‐CoV‐2 infections	[68]
SARS‐CoV‐2 IgA	43^c)^–201^d)^ (AU‐arbitrary units)	SARS‐CoV‐2 infections	[69]

^a)^ Mean values;

^b)^ Median;

^c)^ Control condition;

^d)^ Disease condition;

^e)^ Stage I of disease;

^f)^ Advanced stage of disease.

Active transport and passive diffusion either transcellular or paracellular are normally the biological pathways for the biomarkers to enter saliva.^[^ ^70^ ^]^ The antibodies IgA and IgG form a first‐line immune barrier in the oral cavity against intruding pathogens. IgA appears in saliva via active transport from secretory cells. IgG is believed to enter saliva by passive means through the gingival crevicular fluid.^[^ ^2^ ^]^ Variations of these two antibodies are indicative of viral exposure in saliva tests.^[^ ^58^, ^68^, ^69^ ^]^ Particularly, in cases of SARS‐CoV‐2 infections, the levels of IgG were found in good correlation with the levels in serum.^[^ ^68^ ^]^ The findings denoted the potential of salivary IgG for monitoring the immune response to systemic infections.

C‐reactive protein (CRP) is a marker of systemic inflammation whose levels measured in saliva correlate well to blood levels.^[^ ^71^ ^]^ This protein is acknowledged to be an important risk marker of cardiovascular disease. Recent studies have shown the value of salivary CRP as a confirmatory biomarker of acute myocardial infarction as well as a predictive biomarker of acute lung inflammation in chronic lung disease patients.^[^ ^57^, ^64^ ^]^ Nevertheless, CRP is presented in saliva at concentration values at least one thousand times lower than CRP measured in the blood, making its detection in saliva challenging. Troponins are another class of proteins involved in systemic events. The cardiac troponin I and T (cTnI and cTnT, respectively) when targeted by high‐sensitivity assays have an important role in the early and accurate identification of acute coronary syndrome and myocardial infarction.^[^ ^60^, ^114^ ^]^ These cardiac biomarkers have been detected at a few picograms per milliliter (pg mL^−1^) concentrations in saliva from healthy subjects and tens to hundreds of pg mL^−1^ in samples from diagnosed patients. In addition, the N‐terminal pro‐brain natriuretic peptide (NT‐proBNP), which is the gold standard biomarker for heart failure monitoring, has also been identified in saliva samples although at concentrations (minimum detection of 1 pg mL^−1^) difficult to be accurately analyzed by current biosensors.^[^ ^72^ ^]^

Cytokines are largely produced in the oral cavity and therefore are often associated with oral pathologies. Elevations of interleukins (IL)‐1*β*, IL‐6, TNF‐*α*, and matrix metalloproteinases (MMPs) were identified in periodontitis and hold great potential as biomarkers of diagnosis for this disease.^[^ ^45^, ^46^, ^52^, ^55^ ^]^ IL‐6, IL‐8, and TNF‐*α* have exhibited an area under the curve (AUC) greater than 0.8 for the diagnosis of oral squamous cell carcinoma compared with controls.^[^ ^73^ ^]^ Other reports have suggested a correlation between cytokines and systemic diseases. For instance, elevations of salivary MMP‐9 were statistically significant in primary Sjögren´s syndrome, a systemic autoimmune disorder.^[^ ^56^ ^]^ Levels of salivary IL‐8 were found to have increased significantly in patients with bowel diseases and with muscle and joint diseases.^[^ ^74^ ^]^ IL‐19 has been implicated in systemic inflammatory disorders and has been associated with asthma severity being a potential biomarker for therapy response.^[^ ^75^ ^]^

Besides oral cancer, salivary proteins have also been implicated in lung cancer, breast cancer, and gastric cancer. A panel of three proteins, haptoglobin, zinc‐a‐2‐glycoprotein, and calprotectin, has exhibited an AUC of 0.9 for the detection of lung cancer in comparison with negative controls.^[^ ^76^ ^]^ Clinical sensitivity and specificity of nearly 90% were achieved with this proteomic biomarker panel. In other work, the proteins cystatin B, triosephosphate isomerase, and deleted in malignant brain tumors 1 protein were found to differentiate gastric cancer patients from controls with a detection accuracy of 0.93.^[^ ^54^ ^]^ These reports have also elucidated the need to measure multiple biomarkers in parallel for one diagnosis. Proteomics using mass spectrometry is typically exploited for the discovery of biomarker panels, whereas ELISA or Luminex assays are used to confirm the levels of candidate protein biomarkers in the biofluid.^[^ ^53^, ^66^, ^76^ ^]^

The following sections of this review mention other protein biomarkers in connection to targets of newly developed saliva‐based protein biosensors. Trends in the area of salivary protein biomarkers encompass the discovery of new marker panels with enhanced detection accuracy and further validation of the identified biomarkers in large‐scale clinical trials.^[^ ^67^, ^77^ ^]^

### 1.3 Electrochemical Sensing Method

**Figure 1:**
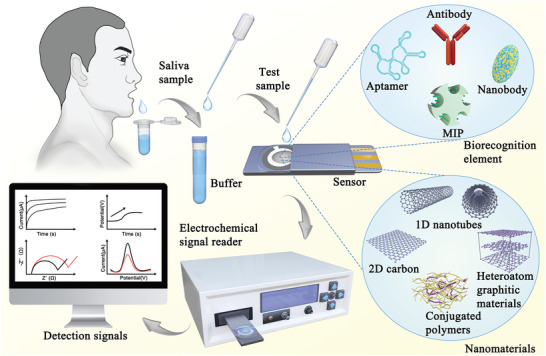
Illustration of electrochemical detection of protein biomarkers in saliva by the nanomaterial‐based biosensor.

Due to the unique characteristics of the electrochemical sensor for POC settings, this type of sensor has been widely used in the sensitive and specific detection of salivary biomarkers.^[^ ^34^, ^35^, ^36^, ^37^, ^38^, ^39^, ^40^, ^41^, ^42^, ^43^, ^44^, ^78^ ^]^ In addition to its promising analytical performance, inherent compactness, low cost, and non‐complicated operation, the miniaturized electrochemical sensor may allow direct detection in saliva with minimal or no addition of extra electrolytes. Compton´s group is a pioneer in electrochemical analyses of saliva samples by exploiting the relatively strong ionic strength of saliva (50–100 mM).^[^ ^79^, ^80^ ^]^ In this case, molecules with redox properties may undertake direct oxidation or reduction on the miniaturized electrode and be measurable by the sensor. However, most proteins identified as chronic or infectious disease biomarkers are electrochemically inert. To realize their detection, the redox probes are used as either label conjugates of biorecognition elements or as a reagent in the solution and subsequently combined with electrode surfaces modified with functional materials.^[^ ^11^, ^32^ ^]^ The roadmap concept of electrochemical detection for protein biomarkers in the saliva is depicted in **Figure** **1**.

#### 1.3.1 Biorecognition Element

In the electrochemical immunoassay, the binding of the protein marker with the respective antibody induces changes in the electrode response typically by variations in voltage, current, or impedance. Antibodies are specific recognition elements often used in saliva‐based biosensors. The antibody can modify the surface of the nanomaterial on the electrode surface, and the variation of the signal is induced either by the complex protein‐antibody directly^[^ ^81^ ^]^ or enhanced by a secondary antibody labeled with an enzyme catalyzing electron donor reactions.^[^ ^82^ ^]^ Oligonucleotide (or peptide) aptamers are also common recognition probes for protein biomarkers. Aptamers benefit from simple chemical synthesis and high stability, are easily tuned in size and conformation, and possess a less complex chemical structure compared with antibodies.^[^ ^83^, ^84^ ^]^

Nanobodies are an emerging class of bio‐receptors with considerable interest in electrochemical protein biosensors.^[^ ^85^, ^86^ ^]^ Nanobodies are variable domains of antibodies, with smaller sizes and higher affinity to targets compared with conventional antibodies. Besides the high binding specificity and high stability, the nanobody possesses an improved solubility and its lower dimensions enable the detection below the Debye lengths.^[^ ^87^ ^]^ “Artificial antibodies” or molecularly imprinted polymers (MIPs) are also reported in recent electrochemical biosensors. MIPs use the target proteins as templates for synthesizing rigid tridimensional (3D) polymer structures around the binding sites of the protein.^[^ ^14^ ^]^ Following the extraction of the template, the target protein is detected by rebinding to the MIP containing empty binding sites. MIPs may comprise conducting polymer structures and may form electroactive films on the top of the electrodes for the transduction of the signal.^[^ ^88^ ^]^

Specific detection of salivary protein has also been conducted with no use of biomolecular probes. Cascade enzymatic reactions involving the hydrolysis of the target and subsequent activation of redox probes have been proposed as one strategy for the specific detection of *α*‐amylase.^[^ ^89^ ^]^ Moreover, functional chemical groups expressed on the nanomaterial surface can be used for the recognition of free terminals of the target protein in the presence of activating agents. For the activation, the immobilization yields of carbodiimide hydrochloride/*N*‐hydroxy succinimide (EDC/NHS) chemistry for instance may allow a strong linkage between the functionalized nanomaterial and the target.^[^ ^6^ ^]^

Biomolecular probes or functional chemical groups are mainly used to modify the working electrode of the sensor. Alternatively, or in addition, the biomolecular probe or the activating chemical groups may functionalize nanostructures in the solution as signal enhancers to the electrochemical sensor.^[^ ^39^, ^90^ ^]^

#### 1.3.2 Sensing Technique

The electrochemical sensor provides sensitive and fast signal transduction of the protein‐receptor binding events. Various techniques of electrochemical sensing have been employed in protein detection from saliva samples, including potentiometry, amperometry, differential pulse voltammetry (DPV), square wave voltammetry (SWV), electrochemical impedance spectroscopy (EIS), and electrochemical capacitance.

Amperometric sensors measure the current response between a working electrode (WE) and counter‐electrode (CE) caused by redox reactions triggered by the recognition of the target analyte.^[^ ^82^ ^]^ The amperometric response is often measured as a function of applied potential (either fixed or swept) or as a function of time upon applying a voltage pulse (chronoamperometry). In potentiometric sensors, the electrochemical potential measured between the WE and reference electrode (RE) varies upon immobilization of the analyte onto the WE surface.^[^ ^91^ ^]^ The measurement is conducted with no current present. Impedimetric sensing is related to the changes in conductance and capacitance at the interface of the WE to the electrolyte. In this case, the specific recognition of the analyte cause variations in the interfacial impedance.^[^ ^92^, ^93^ ^]^ EIS is a representative impedimetric technique involving the measurement of changes in either charge transfer resistance or electrode interface capacitance due to protein‐receptor binding. In EIS electrode currents are measured following the application of an AC signal of varied frequency. The impedimetric measurement can also be done with constant frequency probing variations of dielectric properties at the electrode–electrolyte interface.^[^ ^94^ ^]^ In voltammetric biosensors, the detection of analytes is normally reflected as variations of peak current as a function of applied potentials.^[^ ^37^, ^95^ ^]^ The technique of DPV probes electron transfer from and to electrodes using small pulses whose potential is increased on a linear ramp. The current of detection is measured as the difference between values at two time points, before the application of the pulse and at the end of it. Similar to DPV, SWV obtains the detection (peak) signal by determining the difference between the current value measured at the end of a forward square‐wave pulse and the value at the end of the returning square pulse.

Electrolyte‐gated transistors (EGTs) are increasingly popular in sensitive protein detection.^[^ ^96^ ^]^ EGTs have a similar structure and operation mode to the conventional MOSFETs—metal‐oxide‐semiconductor field‐effect transistors (FETs). While the MOSFET uses a dielectric material for the gate, the EGT uses an electrolyte as the gate dielectric. In EGT the sensor response is controlled by the movement of ions in the electrolyte which occurs following the application of a gate voltage. Depending on the permeability of the EGT channel to the ions of the electrolyte, the transistor acts as an “electrical double‐layer transistor” (EDLT) or as an “electrochemical transistor” (ECT).^[^ ^97^, ^98^ ^]^ The former is the impermeable one in which the gate controls the channel current via a mechanism of capacitive field effect at the channel‐electrolyte interface. In the “electrochemical transistor” the channel is redox‐active and events of doping/dedoping occur upon injection of ions from the electrolyte. For sensing purposes, the EGT is compatible with both amperometric and potentiometric signal transduction.^[^ ^99^ ^]^

Photoelectrochemical (PEC) sensors are also an advanced generation of electrochemical sensors. The PEC sensor has the unique characteristic that the excitation signal (potential) is of a different energy form (optical) than that of the detection signal (electrical). This endows the sensor with enhanced sensitivity and low background noise.^[^ ^83^, ^90^ ^]^ In PEC sensing the photocatalytic properties of semiconductor electrodes are exploited to obtain a current or voltage response, and this response is either enhanced or hindered by the assay involving the binding of the target protein to the electrode surface.^[^ ^100^ ^]^

To maximize the performance of each sensing technique, researchers have made use of the high catalytic efficiency, the large reaction area, and the great biocompatibility of nanomaterials or their related nanocomposites.^[^ ^101^ ^]^ The merits of nanomaterials equip the electrochemical sensor with superior sensitivity, low detection limit, high biosensor stability, and fast response.

This review reports and discusses the latest developments of electrochemical sensors in saliva‐based detection targeting disease‐signaling protein biomarkers. The synergy of electrochemical techniques, nanomaterials, and specific recognition probes is highlighted. Other reviews, namely the recent works of Kaya et al.^[^ ^102^ ^]^ and Mostafa et al.,^[^ ^103^ ^]^ have shed light on electrochemical sensors for biomarkers of various cancers. In these works, the focus is scattered over various biofluid types and analytes, including transcriptomic and metabolic markers. Mani et al.^[^ ^78^ ^]^ discussed the progress of electrochemical sensors with a dispersed focus on drugs, toxins, proteins, and pathogens. Campuzano et al.^[^ ^104^ ^]^ reviewed affinity‐based saliva biosensors before 2017. The present review highlights the advances in electrochemical protein biosensors within the latest 5 years.

## 2 Electrochemical Sensors for Salivary Protein Biomarkers

The following section is divided according to the types of electrochemical sensors that recently emerged in the literature. The discussion of the recent developments in each type of electrochemical sensor includes a critical overview of the strengths and pitfalls of each type. Representative works of each category of sensor accompanied by main performance metrics are described in **Table** **2**.

**Table 2:** Overview of recently developed electrochemical sensors for disease‐signaling protein biomarkers in saliva

Biosensor type	Sensor material	Recognition element	Target	Detection range	Limit of detection	Comment	Ref.
Amperometric/ chronoamperometric	Gold WE	Antibody	TNF‐*α*	1–30 pg mL^−1^	1 pg mL^−1^	Detection in 5 s after incubation with HRP‐labelled secondary antibody and following the addition of TMB substrate.	[34]
Amperometric/ chronoamperometric	Screen‐printed carbon electrode	Antibody	SARS‐CoV‐2 S1 protein	0.5–5 ng mL^−1^	0.15 ng mL^−1^	Incubation with a secondary antibody and addition of a third HRP‐labelled antibody. Detection with a portable analyzer.	[105]
Amperometric/ chronoamperometric	Screen‐printed gold electrode	Aptamer	ODAM	0–15 nM	1 nM	Recognition by a primary aptamer immobilized on electrode and detection by HRP‐linked secondary aptamer. Results were displayed on a smartphone.	[106]
Amperometric/ chronoamperometric	Nanostructured gold coating	Antibody on a DNA linker	SARS‐CoV‐2 S1 protein	—	1 pg mL^−1^	Reagent‐free sensor with ferrocene attached to a DNA linker. Probe binding with protein causes hydrodynamic drag on the sensor affecting the kinetics of the sensor response.	[107]
Potentiometric	Nano‐rough gold film	Molecularly imprinted substrate	SARS‐CoV‐2 and MERS S proteins	>10^2^–10^6^ pg mL^−1^	100 pg mL^−1^	Target proteins used as template molecules fitted conformally on the concave nanostructures of a gold film.	[108]
Impedimetric/EIS	Nanostructured Y_2_O_3_ coating	Antibody	CYFRA‐21‐1	0.01–50 ng mL^−1^	0.01 ng mL^−1^	Y_2_O_3_ nanoparticles enhanced electrode biocompatibility, charge transfer efficiency, and surface‐to‐volume ratio.	[109]
Impedimetric/EIS	Organic polythiophene‐based coating	Antibody	IL‐1*β*	0.01–3 pg mL^−1^	3 fg mL^−1^	Organic nano‐coating enhanced binding sites for antibody immobilization and improved charge transfer efficiency of electrodes.	[110]
Impedimetric/EIS	MWCNT‐AuNP nanocomposite	Antibody	DJ‐1	4.7–4700 fg mL^−1^	0.5 fg mL^−1^	Nanocomposite improved the catalytic activity. MWCNT‐AuNP facilitates electron transfer between antibody and electrode.	[111]
Impedimetric/EIS	Gold WE	Aptamer	SARS‐CoV‐2 S1 protein	4–44 000 fM	1 fM	Dimeric DNA aptamer immobilized on a thiolated gold electrode with superior affinity to S proteins. Detection under 10 min.	[112]
Impedimetric/EIS	Screen‐printed gold electrode with nano‐porosities	Molecularly imprinted polymer	SARS‐CoV‐2 RBD	2–40 pg mL^−1^	0.7 pg mL^−1^	Immobilization of target protein in a MIP film hindered diffusion of the redox probe on the electrode surface. Nano‐porosities enhanced active surface area. Detection in 20 min.	[113]
Impedimetric/EC capacitance	Al/Si/SiO_2_/Si_3_N_4_	Antibody	TNF‐*α*	1–30 pg mL^−1^	1 pg mL^−1^	Measurement of EC capacitance changes by protein‐antibody binding complexes on a high dielectric material (film, 100‐nm thick).	[94]
Voltammetric/DPV	ZnO‐rGO nanocomposite	Antibody	IL‐8	10^−4^–5 ng mL^−1^	≈50 pg mL^−1^	Nanocomposite film exhibited quasi‐reversible electrochemical characteristics in the cyclic voltammogram for Zobell´s solution. Tests in undiluted saliva samples.	[114]
Voltammetric/DPV	Nitrogen‐doped rGO	Aptamer	cTnI	1–10^5^ pg mL^−1^	1 pg mL^−1^	Decreased peak current by the complex DNA aptamer‐cTnI. rGO provided a large surface area and its functionalization by py‐PEG allowed optimizing bioreceptor density.	[115]
Voltammetric/DPV	MWCNTs	Antibody	IL‐1*β*	10–1200 pg mL^−1^	5.2 pg mL^−1^	Azide‐functionalized MWCNTs bound to ethynylated primary antibody by electro‐click chemistry. Secondary antibody labeled with AP‐Strep label for enzymatic redox.	[116]
Voltammetric/DPV	AuNPs coupled to magnetic particles	Peptide sequence	SARS‐CoV‐2 RBD	—	0.35 ag mL^−1^	Both AuNPs and magnetic particles were modified with ACE2 peptide to specifically bind RBD. Detection by reduction of [AuCl_4_]^−^ to Au after the addition of HCl solution.	[117]
Voltammetric/DPV	Bi_2_WO_6_/Bi_2_S_3_ heterostructure	Antibody	SARS‐CoV‐2 N protein	0.01–1 pg mL^−1^	3 fg mL^−1^	Bi_2_WO_6_/Bi_2_S_3_ was used as the sensor platform coated by primary antibody. Signal enhancement by g‐C_3_N_4_/Au/WO_3_ composite as a label of secondary antibody.	[118]
Voltammetric/SWV	Au nanowires	Antibody	CRP	8–140 fg mL^−1^	4 fg mL^−1^	Arrays of nanowires improved electron transfer efficiency for [Fe(CN)_6_]^3‐/4−^, and enhanced the surface area and the biocompatibility to adsorbed biomolecules.	[119]
Voltammetric/SWV	Nanoporous anodic aluminum oxide on LEGE	Aptamer	SARS‐CoV‐2 RBD	2.5–40 ng mL^−1^	0.8 ng mL^−1^	Nanoporous membrane coated with AuNPs and functionalized with DNA aptamer. The aptamer‐RBD complex hindered access of an electrode to a redox probe.	[120]
Electrolyte gated transistor/EDLT	Carbon nanofibers	Antibody	Nesfatin‐1	10–10^6^ fM	10 fM	A FET channel was made of multi‐pore carbon nanofibers. Analyte binding to the channel‐immobilized antibody led to variation in channel charge transport.	[121]
Electrolyte gated transistor/EDLT	rGO	Aptamer	HPV‐16 E7 protein	—	100 pg mL^−1^	rGO prepared onto silanized interdigitated electrodes. Change of conformation of an RNA aptamer by binding with protein altered current output. Limit of detection in a buffer.	[122]
Electrolyte gated transistor/ECT	Poly(3‐hexylthiophene‐2,5‐diyl)	Antibody	CRP	—	≈13 molecules per 100 µL	An organic semiconductor is used as the FET channel. Gate functionalized with antibody. Detected shifts in the transfer *I*–*V* curves due to analyte binding to the respective antibody.	[123]
Electrolyte gated transistor/ECT	PEDOT:PSS	Spike protein, His Tag	SARS‐CoV‐2 IgG protein	10–10^8^ fM	10 fM	The surface potential of the FET gate varied with the specific binding of IgG on the gate and led to shifts in the *I*–*V* transfer curves. Assay time of 5 min including incubation.	[124]
Photoelectrochemistry	Pd NPs/g‐C_3_N_4_‐S/SrTiO_3_ nanocomposite	Antibody	SARS‐CoV‐2 S1 protein	1–10^6^ fg mL^−1^	1 fg mL^−1^	The binding of S1 proteins to antibodies decreased the efficiency of photon‐to‐current conversion in PdNPs/g‐C_3_N_4_‐S/SrTiO_3_ composite in contact with an electrolyte.	[125]
Photoelectrochemistry	Ti_3_C_2_T*_x_*/NiWO_4_ nanocomposite	Antibody	Prostate‐specific antigen	1.2–0.18 × 10^12^ fg mL^−1^	0.15 fg mL^−1^	NiWO_4_ nanoparticles and Ti_3_C_2_T*_x_* sheets formed a heterostructure with fast interfacial charge transfer kinetics. Detection is conducted by inhibition of photocurrent.	[126]
Photoelectrochemistry	CdS QDs/g‐C_3_N_4_ nanocomposite	Aptamer	SARS‐CoV‐2 RBD	0.5–32 nM	0.12 nM	Immobilization of RBD protein by a DNA aptamer caused hindrance to mass transport of redox probe to the nanocomposite surface, reducing photocurrent response.	[127]

Abbreviations: HRP—Horseradish peroxidase; TMB—Tetramethylbenzidine; ODAM—Human odontogenic ameloblast‐associated protein; MERS—Middle‐East respiratory syndrome coronavirus; EIS—Electrochemical impedance spectroscopy; Y_2_O_3_—Yttrium oxide; MWCNT‐AuNP—Multiwalled carbon nanotube‐gold nanoparticle; EC—Electrochemical; RBD—Receptor‐binding domain; Al—Aluminum; SiO_2_—Silicon dioxide; Si_3_N_4_ silicon nitride; ZnO—Zinc oxide; rGO—Reduced graphene oxide; cTnI—Cardiac troponin I; AP‐Strep—Alkaline phosphatase‐streptavidin conjugate; ACE2—Human angiotensin‐converting enzyme 2; Bi_2_WO_6_/Bi_2_S_3_—Bismuth tungstate/bismuth sulfide composite; g‐C_3_N_4_/Au/WO_3_—Graphitic carbon nitride sheet decorated with AuNPs and tungsten trioxide composite; LEGE—Laser engraved graphene electrode; EDLT—Electrical double‐layer transistor; HPV—Human papillomavirus; ECT—Electrochemical transistor; PEDOT:PSS—Poly(3,4‐ethylenedioxythiophene)−poly(styrenesulfonate); Pd NPs/g‐C_3_N_4_‐S/SrTiO_3_—Palladium nanoparticles/sulfur‐doped carbon nitride/strontium titanate; CdS QDs—Cadmium sulfide quantum dots.

### 2.1 Amperometric Sensors

Amperometry is one of the primary forms of biosensing since the demonstration of the amperometric measurement of glucose by Leland C. Clark.^[^ ^128^ ^]^ For protein analysis in saliva, amperometric sensing is commonly conducted using functionalized Au electrodes. Salivary TNF‐*α* has been detected by an Au WE functionalized with a TNF‐*α*‐capture antibody.^[^ ^34^ ^]^ The amperometric response was obtained from the redox reaction of tetramethylbenzidine (TMB) on the Au electrode catalyzed by the horseradish peroxidase (HRP). The HRP enzyme is commonly utilized as a label for a secondary antibody that recognizes the protein target. In this work,^[^ ^34^ ^]^ chronoamperometry (CA) was employed to record the differences in the detection signal among the tested TNF‐*α* samples ranging from 1 to 30 pg mL^−1^ in concentration. A limit of detection (LOD) of 1 pg mL^−1^ protein was reported. Although the CA measurement was executed in only 5 s, the total assay time exceeded 1 h.

The Au electrode can be modified with micron or nano‐sized particles to increase the surface area and enhance electrode conductivity. TNF‐*α* was detected in artificial saliva samples by a CA immunoassay employing an Au electrode with surface modification by magnetic particles.^[^ ^129^ ^]^ TNF‐*α*‐capture antibody was coated on the magnetic particle surface. As a result, the LOD of the sub‐micron rough Au electrode has improved to 0.3 pg mL^−1^, corresponding to one order of magnitude lower than the planar Au electrode.^[^ ^34^ ^]^

**Figure 2:**
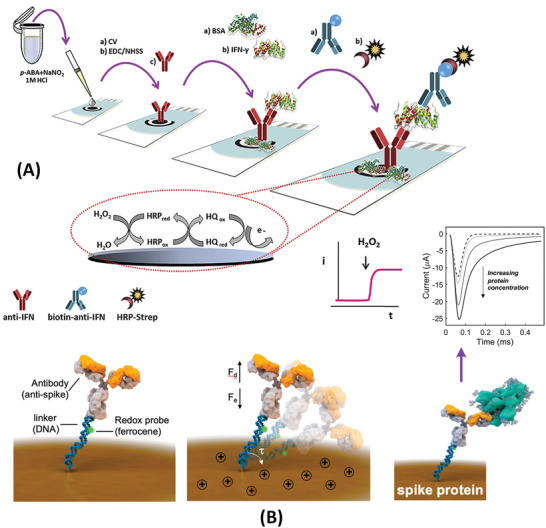
Amperometric sensors for detection of salivary protein biomarkers. A) Carbon‐electrode‐based immunoassay for IFN‐*γ* coupled to chronoamperometry. Reproduced with permission.^[^ ^130^ ^]^ Copyright 2020, Elsevier. B) Reagent‐free electrode‐tethered immunosensor. Inset shows different current decay due to different protein biomarker concentrations. Reproduced with permission.^[^ ^107^ ^]^ Copyright 2021, American Society of Chemistry. Abbreviations: EDC/NHSS—1‐ethyl‐3‐(3‐dimethylamino‐propyl) carbodiimide/*N*‐hydroxy sulfosuccinimide; HQ—hydroquinone; HRP‐Strep—horseradish peroxidase‐labeled streptavidin.

Screen‐printed electrodes (SPEs) are a low‐cost alternative to the Au electrodes for protein assays exhibiting no loss of analytical performance. Carbon‐based SPEs were used in amperometric assays for salivary interferon‐gamma (IFN‐*γ*).^[^ ^130^ ^]^ This SPE‐based sensor has also handled a “sandwich‐type” immunoassay, in which the IFN‐*γ*‐capture antibody modified with the surface of the SPE and a secondary antibody labeled with HRP completed the assay (see **Figure** **2**A). The amperometric response was obtained through HRP/hydroquinone/H_2_O_2_ redox cycling on the WE. The LOD of this SPE‐based assay was around 1 pg mL^−1^ while the detection range was from a few pg mL^−1^ to 2000 pg mL^−1^. Of remark, the assay has handled undiluted samples of human saliva by utilizing WE surface blockage with bovine serum albumin (BSA). Screen printing can also be applied to make low‐cost Au electrodes and turn them more amenable to use in resource‐poor settings. An Au‐based SPE was fabricated for CA immunoassays analyzing a periodontal disease protein.^[^ ^106^ ^]^ Contrary to the amperometric sensors above‐reviewed, this SPE was coupled with aptamers used as the biorecognition elements. A first “capturing” aptamer has coated the SPE surface and a second “detecting” aptamer labeled with HRP recognized the target protein and completed the assay. The redox reaction of TMB catalyzed by the HRP label was exploited for the CA measurements. The assay times were still over 1 h.

Besides modifying the surface of the Au electrode with micro‐ or nanoparticles, the Au electrode can be patterned with nanoscale features by adding a second Au layer via electrodeposition. The nanostructured Au electrode has exhibited good electronic properties and it was used in a new concept of amperometric tethered sensors.^[^ ^107^, ^131^ ^]^ The tethered sensor consisted of immobilizing a biomolecular complex made of a thiolated DNA linker and a detection antibody atop the nanostructured Au electrode. A redox reporter (ferrocene) was tethered to the DNA linker and mediated the detection via its redox reaction on the electrode surface. The tethered sensor has detected SARS‐CoV‐2 spike (S) protein with a LOD as low as 1 pg mL^−1^. The assay is shown in Figure 2B. By applying a positive potential (+0.5 V), the biomolecular complex is brought into contact with the electrode surface occurring the oxidation of ferrocene. Using the CA measurement, it can be observed a slower decay in the current response due to the hydrodynamic drag force (*F*_d_) that balances the force induced by the electric field (*F*_e_). The lower the concentration of protein the faster the current decays. Each analyte measurement took only 5 min, and tests were conducted with clinical saliva samples revealing minimal interference from the sample matrix on the amperometric response.

In summary, planar and nanostructured Au electrodes and SPEs are still predominant in amperometric sensors targeting protein detection. These sensors typically utilize HRP as a label of detection antibody and exploit its catalytic activity to redox reagents in solution. This type of assay exhibits potential for analyzing minimally processed saliva samples by coating the electrode surface with BSA; on the other side, this coating can minimize the electroactive area to some extent. The advent of tethered sensors with surface‐linked redox reporters creates an opportunity to further simplify the amperometric sensor, enabling a faster test besides making it non‐reagent based. Incubation with the saliva sample becomes the single step of the assay, thereby facilitating the realization of a fully automated sensor.

#### 2.1.1 Pros and Cons of Amperometric Sensors

The advantages of amperometric sensors are 1) the simplicity of the sensor with easy integration on‐chip and potentially low cost, 2) the fast signal transduction and rapid assaying with tethered sensors, and 3) the possibility of detecting protein markers in minimally prepared saliva samples. The disadvantages of this electrochemical technique include: 1) The LODs are still reported in the pg mL^−1^ level; 2) the activity of enzyme‐based amperometric sensors is affected by variations in pH and temperature. This can be of concern upon rapid introduction of an unprepared biological specimen; 3) the assay times are relatively long with the introduction and incubation of labeled detection antibody which follows the analyte diffusion and incubation on the electrode surface.

### 2.2 Potentiometric Sensors

**Figure 3:**
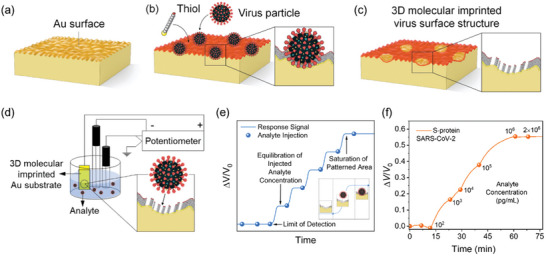
Potentiometric sensor for saliva‐based protein detection exploring a nanostructured working electrode. a) Nano‐rough gold (Au) surface on a silicon substrate. b) Imprinting of template molecules (hereby viral particles; the process is the same as spike proteins alone) on the thiol‐Au surface. c) Removal of template molecules forming an imprinted thiol layer. d) Potentiometric sensing of the analyte. e) Potentiometric response when the analyte is added (*V*_0_, starting baseline voltage). f) Detection of target protein biomarker. Reproduced with permission.^[^ ^108^ ^]^ Copyright 2022, American Chemical Society.

In saliva, the potentiometric sensor is widely exploited for the detection of inorganic indicators such as pH or ions such as salivary thiocyanate.^[^ ^78^ ^]^ For protein analysis, the developments of potentiometric sensors have been focusing on exploiting the readout properties of nanostructured electrodes. Potentiometric sensors incorporating a nanostructured Au coating on silicon and a MIP recognition element were proposed for the detection of various salivary biomarkers.^[^ ^108^, ^132^ ^]^ Hereby, the target protein is used as the template for a 3D imprinting technique applied to an Au coating with nano‐roughness. The template molecules are adsorbed onto the concave areas of the Au coating, and thiols are crystallized around the template by reacting with Au and forming the binding sites of the sensor. After the removal of the template, the imprinted thiol layer binds specifically to the target proteins, and the binding complexes cause variation in the open‐circuit potential of the sensor. This type of potentiometric sensor is shown in **Figure** **3**. The performance of detection is controlled by the roughness of the Au coating whose concave structures shall match the size of the bio‐target. Smaller molecule sizes would demand a smoother Au surface at the nanoscale, and it is the polishing grade of the silicon substrate surface that defines the roughness of Au. The sensor was demonstrated for saliva samples and exhibited a detection time shorter than 5 min. Its analytical performance in saliva has so far been shown for viral particles, namely Zika virus or Dengue virus,^[^ ^132^ ^]^ and protein markers such as SARS‐CoV‐2 S proteins^[^ ^108^ ^]^ and cancer embryonic antigen.^[^ ^133^ ^]^

Based on carbon ink electrodes and utilizing no biomolecular probes, another approach of the potentiometric sensor was realized for *α*‐amylase, a common disease marker analyzed from human saliva.^[^ ^134^ ^]^ The carbon electrodes were part of reagent strips containing two separate channels under different pH (one under alkaline pH and the other under neutral pH). The target *α*‐amylase can hydrolyze starch into maltose;^[^ ^89^ ^]^ subsequently, under the alkaline solution, the generated maltose reduces Fe(CN)_6_]^3−^ to [Fe(CN)_6_]^4−^. The reaction does not occur in the neutral condition. Therefore, the difference in the ion ratio between two parallel channels can be explored for obtaining an electrical potential difference between two electrodes, thereby indirectly detecting *α*‐amylase. This potentiometric sensor can provide the result in 2 min and measures the target at the physiologically relevant concentrations.

In conclusion, the lower utilization of the potentiometric sensor for protein analysis compared to other electrochemical techniques such as the amperometric sensor or impedimetric sensor is explained by the difficulty of adopting potentiometric measurements in affinity‐based sensing.^[^ ^91^ ^]^ Despite this issue, the combination of MIPs and nanostructured Au electrodes offers potential for a class of potentiometric sensors with utility in biomarker detection cases demanding fast responses. The concept is suitable for mass production.

#### 2.2.1 Pros and Cons of Potentiometric Sensors

The strengths of the potentiometric sensor are 1) the detection in a few min, 2) the simplicity of the sensor, and 3) the use with no sample treatment. The main drawbacks include 1) low detection sensitivity, 2) limited linear response, 3) susceptibility to variations of pH and temperature, and requires frequent calibrations, and 4) high requirement for a stable and accurate reference electrode.

### 2.3 Impedimetric Sensors

Impedance sensing in saliva has been realized using unstructured and nanostructured electrodes made of metal oxides, carbon, and Au. Moreover, it is denoted an increasing use of graphitic nanomaterials in electrochemical impedance sensing.

Indium tin oxide (ITO) is a widely used material in impedance sensors despite its limitations in achieving a prominent charge transfer efficiency.^[^ ^104^, ^110^ ^]^ The ITO electrode has the advantages of being low‐cost and providing a surface highly compatible with a variety of chemical methods for the immobilization of biorecognition probes.^[^ ^93^, ^135^ **^]^** Unstructured ITO is often functionalized with self‐assembled layers (SAMs) for the attachment of antibodies specific to the target protein. After incubation of the protein (typically taking more than 30 min), the [Fe(CN)_6_]^3−/4−^ redox probe is added to the electrode surface to obtain the EIS signal which varies with the interaction between the protein and the immobilized antibodies. This approach was used to detect IL‐1*β* on ITO electrodes modified with carboxyl‐activated 6‐phosphohexanoic acid as the SAM.^[^ ^135^ ^]^ The sensor monitored the resistance of the electrode to electron transfer (*R*_et_) upon redox reactions of [Fe(CN)_6_]^3−/4−^ on the electrode surface. Diffusion and kinetics of [Fe(CN)_6_]^3−/4−^ were hindered on the electrode surface due to increasing concentrations of IL‐1*β*. This is a typical assay methodology to achieve protein detection in EIS biosensors. The EIS detection on the unstructured ITO electrode exhibited a LOD of 7.5 fg mL^−1^ and a detection range of 0.025 to 3 fg mL^−1^ from centrifuged and 20‐fold diluted saliva samples.^[^ ^135^ ^]^

Conductive composite materials are a solution with great potential to further improve the analytical characteristics of the unstructured ITO electrode for impedance detection. A composite electrode made of ITO and a polythiophene derivative conjugated polymer has been proposed to enhance the charge transfer efficiency of the electrode while providing more binding sites for the immobilized of the antibody.^[^ ^110^ ^]^ The synergy of these two effects led to a twofold decreased LOD for IL‐1*β* detection compared with the aforementioned SAM‐ITO electrode. The incubation time and total assay duration were not changed with the use of the composite electrode. In another work,^[^ ^93^ ^]^ a twofold reduced LOD for detecting salivary interleukins was achieved by modifying the surface of ITO with a composite film made of carbon black, poly(glycidyl methacrylate), and polyvinylidene fluoride. Hereby, the interleukin IL‐8 was detected in 50‐fold diluted saliva samples, and the incubation times were still longer than 30 min.

Another class of impedimetric sensors involves the nanostructuring of SPEs. Nanostructured SPEs can be taken as an alternative to ITO composite electrodes to improve charge transfer efficiencies, and enhance biocompatibility for the immobilization of biomolecular probes, in addition to providing an increased surface area. A carbon‐based SPE nanostructured with gold nanoparticles (AuNPs) was demonstrated for the detection of SARS‐CoV‐2 nucleocapsid (N) protein in tenfold diluted saliva samples.^[^ ^136^ ^]^ The sensor was constructed by electrodeposition of the AuNPs on the SPE forming a nanostructured film, followed by surface modification with streptavidin used as a linker to the attachment of a biotinylated antibody. A solution of [Fe(CN)_6_]^3−/4−^ was added to the nanostructured film electrode to obtain the EIS signal following a similar procedure as with the aforementioned ITO‐based impedimetric sensors. The nanostructured Au impedimetric sensor exhibited a LOD of few pg mL^−1^ and good reproducibility thanks to the large catalytic area and highly oriented linking of the detection antibody. In another work,^[^ ^113^ ^]^ an impedimetric sensor was formed by an Au‐based SPE with nano‐sized porosities (peak to valley height of 30.6 nm). The sensor detected the receptor‐binding domain (RBD) of the SARS‐CoV‐2 S protein with a MIP film formed on the porosity valleys of the nanostructured electrode. While the nanoporosities enabled an electrode surface with low background resistance to charge transfer, the interaction of the target RBD with the specific binding sites on the MIP increased substantially *R*_et_ of the electrode in the presence of [Fe(CN)_6_]^3−/4−^. The effect was proportional to the concentration of protein. Of remark, this nanostructured impedimetric sensor measured RBD in twofold diluted saliva samples with an analysis time of 20 min.

**Figure 4:**
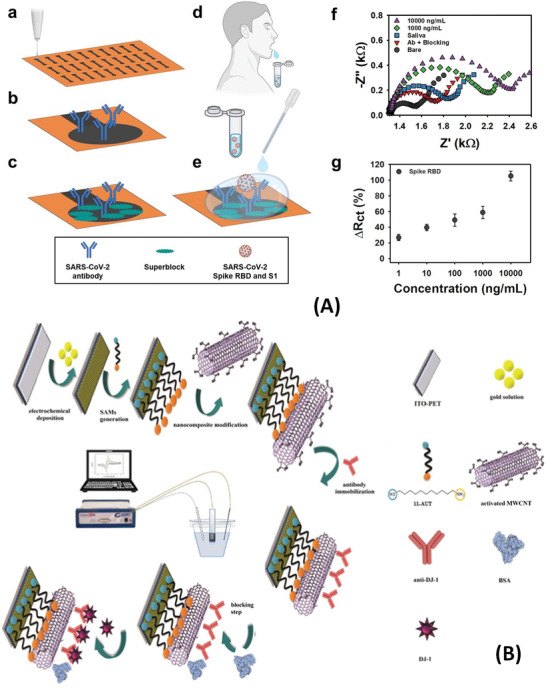
Impedimetric sensors for detection of salivary protein biomarkers. A) Illustration of a graphene‐based EIS sensor with the steps: a) Aerosol jet printing of graphene ink with dispersed nanosheets. b) Immobilization of antibodies. c) Blocking of unmodified graphene areas against non‐specific adsorption. d) Sampling method proposed for the saliva test with the sensor. e) Incubation of the sample containing either S1 protein or RBD). f) Nyquist plot with and with no analyte. g) Charge transfer resistance response (Δ*R*_ct_) due to RBD concentrations in saliva. Reproduced with permission.^[^ ^138^ ^]^ Copyright 2022, IOP Publishing. B) Illustration of a nanocomposite impedimetric sensor made of ITO electrode immobilized with MWCNTs and AuNPs. Reproduced with permission.^[^ ^111^ ^]^ Copyright 2021, Elsevier. Abbreviation: 11‐AUT—1‐amino‐1‐undecanethiol.

Carbon nanotubes and graphene are among the most promising graphitic nanomaterials for EIS sensors due to their efficient electron transfer, high catalytic activity, and low interfacial resistance.^[^ ^102^, ^137^ ^]^ A graphene ink formulation made of exfoliated graphene nanosheets has been used for preparing sensitive EIS sensors. These sensors have targeted SARS‐CoV‐2 S1 protein and RBD in artificial saliva exhibiting a detection range of 1–1000 ng mL^−1^ and LODs of ≈20 pg mL^−1^ and ≈110 pg mL^−1^ for RBD and S1, respectively.^[^ ^138^ ^]^ An overview of the concept is shown in **Figure** **4**A. The approach has benefited from a facile modification of the graphene film electrode by antibody and surface blocking agent. The high‐throughput and inexpensive printing of the electrodes ($3.39 per unit) are also advantages of these graphene‐printed sensors. Nevertheless, [Fe(CN)_6_]^3−/4−^ is still used to generate the EIS response. Analysis time has exceeded 30 min. Multi‐walled carbon nanotubes (MWCNTs) alone or as part of composite electrodes are also promising materials for sensitive impedance detection. A nanocomposite electrode made of MWCNTs and AuNPs was prepared to detect DJ‐1 protein as an important biomarker of Parkinson´s disease and oxidative stress.^[^ ^111^ ^]^ Antibodies specific to DJ‐1 were immobilized on the surface of MWCNTs (Figure 4B), and the composite interface MWCNTs‐AuNPs ensure high charge transfer characteristics. The interaction of the target protein with the immobilized antibodies created variations of both *R*_et_ and electrochemical capacitance of the electrodes in the presence of [Fe(CN)_6_]^3−/4−^. This nanocomposite‐based EIS sensor achieved a remarkably low LOD (0.5 fg mL^−1^); nevertheless, a 10^6^‐fold dilution of saliva was necessary to execute detection in clinical samples with minimal signal interferences.

In conclusion, low‐cost electrodes such as ITO and SPEs are coated with nanostructured materials to decrease the LODs of impedance sensors which can reach fg mL^−1^. The basis of signal amplification is to maximize electron transfer efficiency between the electrode and electrolyte, and hence to reduce the background *R*_et_ before surface‐binding of the protein. Among the reports analyzed, the detection range has seldom surpassed four orders of magnitude either with uncoated metal or carbon electrodes or with those coated with nanomaterials. A detection range of five orders of magnitude was achieved by coating a dimeric DNA aptamer on a thiolated Au electrode.^[^ ^112^ ^]^ This work may reinforce further the fact that the design of the biosensor needs to consider the synergy of electrode materials and properties of biorecognition elements. The analysis times with impedimetric sensors are typically in the order of tens of min and are regulated by the diffusion and incubation of the analyte on the electrode surface. The steric hindrance created by the protein/bio‐recognition probe complexes on the surface reactivity of a redox probe is the basic sensing mechanism in impedimetric sensors. This mechanism often waives the use of a labeled secondary antibody simplifying the assay. Saliva dilution is still the common procedure for preparing the sample for impedance sensing.^[^ ^110^, ^136^ ^]^

#### 2.3.1 Pros and Cons of Impedimetric Sensors

Pros of impedimetric sensors are 1) the compatibility of the sensing technique to use of different bio‐receptors including antibodies, aptamers, and MIPs, 2) the possibility of developing sensitive biosensors from low‐cost electrode surfaces, 3) the wide availability of EIS electrode designs comprising various types of composite materials. The major cons concerning protein detection in saliva encompass: 1) The need for introducing a redox probe in solution to execute the detection; the reagent‐based assay increments one step in the operation of the sensor following incubation with the analyte, 2) the need for more complex data analysis compared to amperometric or impedimetric sensors involving data fitting to equivalent circuits, 3) the limitation of sensing mechanisms to steric hindrance which may restrain the development of strategies to enhance detection sensitivity.

### 2.4 Voltammetric Sensors

DPV and SWV are the most representative voltammetry‐type techniques for protein detection in saliva. Both techniques produce well‐defined peak currents in rapid assays, exhibit high signal‐to‐noise ratios, and require no complex signal processing such as fitting to equivalent circuits as in the case of EIS.

AuNPs have been intensively exploited in DPV^[^ ^117^, ^139^ ^]^ and SWV^[^ ^119^, ^120^ ^]^ sensors due to their high catalytic properties for redox reactions. For analysis of protein biomarkers in saliva, AuNPs have majorly been used in two sensing formats, either as labels of biorecognition probes^[^ ^117^ ^]^ or as modifiers of the metal electrode or composite electrode surfaces.^[^ ^120^ ^]^ A DPV sensor for sIgA was realized by labeling secondary (detection) antibodies with AuNPs. A peak current response was obtained from the electrochemical reduction of the complex [AuCl_4_]^−^ to Au in the presence of diluted acid, and the response was proportional to the amount of sIgA bound to the AuNP‐labeled antibody. The LOD of this DPV assay was in the order of a few ng mL^−1^.^[^ ^139^ ^]^ A similar assay strategy was used in another DPV sensor using AuNPs bound to human angiotensin‐converting enzyme 2 (ACE2) peptide which acted as the bio‐receptor to SARS‐CoV‐2 RBD.^[^ ^117^ ^]^ A remarkably low LOD of 0.35 ag mL^−1^ was achieved with this AuNP‐ACE2 sensor when an extra bio‐recognition probe made of ACE2 labeled with magnetic particles was added to the assay.

**Figure 5:**
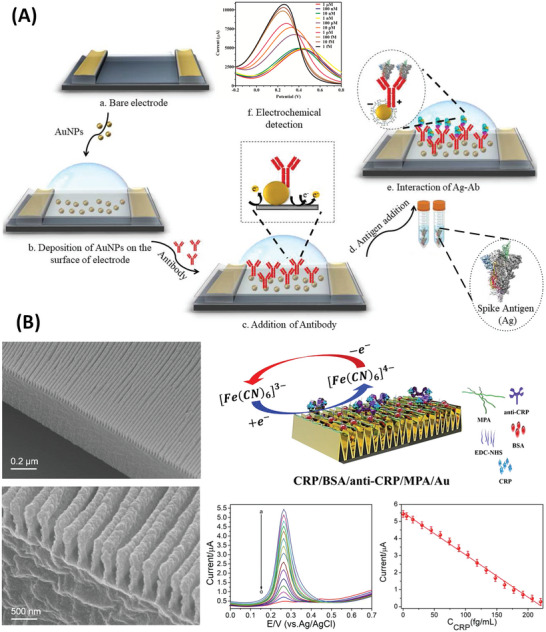
Voltammetric sensors based on Au nanostructures. A) DPV sensor for the detection of SARS‐CoV‐2 S proteins exploiting the catalytic effect and large surface area of AuNPs. Inset (f) demonstrates decreasing in DPV response due to increasing protein concentrations. Reproduced with permission.^[^ ^140^ ^]^ Copyright 2021, Elsevier. B) SWV sensor with Au nanowires for the detection of salivary CRP. Insets of the bottom side of (B) show the SWV response due to increasing CRP concentrations (from “a” to “o”) and the respective calibration curve based on peak currents. Reproduced with permission.^[^ ^119^ ^]^ Copyright 2019, Elsevier. Abbreviation: MPA—3‐mercaptopropionic acid.

AuNPs have acted as modifiers of voltammetric sensors to enhance their conductivity and confer larger catalytic areas (**Figure** **5**A). For a DPV sensor detecting SARS‐CoV‐2 S1 protein in saliva, AuNPs were used to modify the surface of fluorine‐doped tin oxide electrodes.^[^ ^140^ ^]^ The nanoparticles guaranteed a high peak current response in the presence of [Fe(CN)_6_]^3−/4−^, which has decreased with the formation of protein‐antibody complexes atop the AuNPs. For an SWV sensor detecting SARS‐CoV‐2 RBD, AuNPs have modified the surface of a composite electrode made of nanoporous aluminum oxide membranes and graphene.^[^ ^120^ ^]^ The nanoparticles were expressed atop the composite electrode and were bound to thiolated aptamers via Au—S bonds. The SWV was maximum with no presence of analyte indicating the high diffusivity of the nanoporous sensor to a redox probe. By binding of target RBD with the surface‐immobilized aptamer, mass transfer of the redox probe was hindered on the electrode surface, leading to a decreased Faradaic current. Besides offering a large surface area for aptamer binding, AuNPs have also enhanced charge transfer through this nanoporous electrode. The SWV response was obtained from 1:4 diluted saliva samples and the analysis time surpassed 20 min.

The utilization of AuNPs to confer nanostructures on voltammetric sensors is a common procedure; nevertheless, Au in form of other nano‐scale architectures can also be exploited to achieve notable charge transfer efficiencies and enhance peak currents. Au in form of nanowire arrays was synthesized on top of polymer substrates for the detection of salivary CRP at a limit of a few fg mL^−1^.^[^ ^119^ ^]^ The nanowires provided a truly enlarged surface for antibody immobilization and facilitated the redox cycling of [Fe(CN)_6_]^3−/4−^, being responsible for the high catalytic activity of the voltammetric sensor (Figure 5B). Of note, the SWV response of this sensor can be optimized for various bio‐targets by regulating the size and density of nanowires using a nanoimprint lithography process. This SWV sensor achieved three orders of magnitude as the detection range for CRP measured in tenfold diluted saliva samples.

Graphitic nanomaterials have emerged as alternatives to Au nanostructures for sensitive voltammetric sensors. Besides exhibiting naturally high catalytic activity and in the meanwhile good biocompatibility, the graphitic nanomaterials benefit from facile manipulation of their surface and lattice composition, tuning physicochemical properties, and creating a new generation of voltammetric sensors with outstanding peak current responses.

Graphene oxide (GO) with or without atomic and surface modifications has been designed for a variety of DPV and SWV sensors. GO with modification can be used as a coating for common SPEs and glassy carbon electrodes (GCEs). A series of SWV sensors with GO‐coated SPEs and GCEs were developed for SARS‐CoV S1 proteins using antibodies as the biorecognition element. After the addition of an electrolyte, the S1 proteins were quantified in the range of attograms to femtograms per mL from saliva samples pretreated with a lysis buffer.^[^ ^141^ ^]^ This GO‐based SWV sensor demonstrated a diagnostic accuracy of over 90% in positive virus‐infected samples which contrasted with less than 67% achieved with a commercial antigen test kit.

Atomic modification of GO has also been exercised to tune the electrocatalytic properties of this nanomaterial. GO can be transformed into a porous material and doped with different elements to enhance the electroactive area of GO‐coated electrodes. Using this route of GO modification, a nitrogen‐doped GO coating on a GCE electrode was reported for the detection of cTnI in undiluted saliva samples.^[^ ^115^ ^]^ The porous structure of GO enabled a large surface area for the immobilization of a DNA aptamer and guaranteed high DPV peak currents. The interactions of cTnI and the aptamer were measured at cTnI concentrations spanning six orders of magnitude, which indicated the good signal‐to‐noise ratio of the modified GO sensor. Minimal biological interferences in the test of undiluted samples were ensured by a poly(ethylene glycol) coating which was highly compatible with the nitrogen‐doped GO.

Moreover, GO can be used to stabilize metal oxide nanoparticles which also hold promising electrocatalytic properties for voltammetric sensors. Nanocomposites of GO with zinc oxide (ZnO) nanoparticles were prepared for DPV sensors targeting salivary IL‐8.^[^ ^114^ ^]^ Chemical functionalization of the nanocomposite surface with ethanolamine ensured reproducible measurements of IL‐8 in undiluted saliva samples. GO has also stabilized yttria‐doped zirconia nanoparticles exploited as a sensing platform to detect salivary CYFRA‐21‐1.^[^ ^142^ ^]^ These GO‐stabilized DPV sensors exhibited LODs for protein detection in the order of pg mL^−1^.

**Figure 6:**
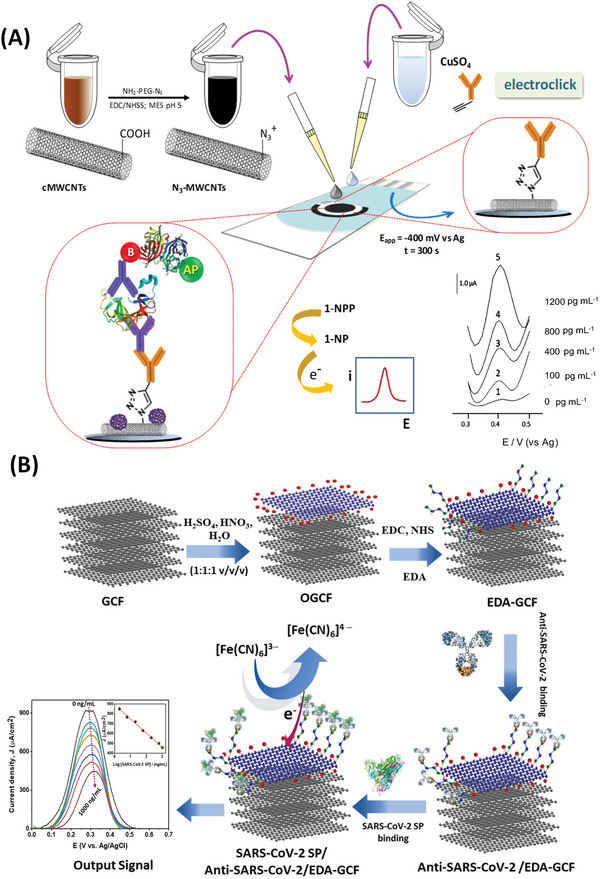
Voltammetric sensors based on graphitic nanomaterials for the detection of salivary interleukin IL‐1*β* and SARS‐CoV‐2 S proteins. A) Screen‐printed electrode modified with MWCNTs. Deposition of MWCNTs was followed by adding CuSO_4_ containing anti‐IL‐1*β* IgG. DPV was measured using 1‐naphthylphosphate (1‐NPP) in presence of an alkaline phosphatase label. Reproduced with permission.^[^ ^116^ ^]^ Copyright 2020, Elsevier. B) Biosensor with oxidized graphitic carbon foil (OGCF) and functionalized with antibody against S1 protein. Inset shows the DPV signals from detecting various protein concentrations causing a decrease in peak current. Reproduced with permission.^[^ ^143^ ^]^ Copyright 2022, Elsevier. Abbreviation: EDA—ethylenediamine.

Besides GO, other graphitic nanomaterials have emerged in the literature for voltammetric sensing. MWCNTs with surface modification with 11‐azide‐3,6,9‐trioxaun‐decan‐1‐amine have significantly improved the peak current response of carbon‐based SPEs.^[^ ^116^ ^]^ This azide‐MWCNTs‐based sensor has detected IL‐1*β* in minimally processed saliva samples. For the assay, a primary antibody was immobilized on MWCNTs via electro‐click chemistry, and a secondary antibody labeled with alkaline phosphatase‐streptavidin was loaded on the electrode after incubation of IL‐1*β*. Alkaline phosphatase catalyzed the conversion of 1‐naphthyl phosphate to 1‐naphthol leading to DPV currents (**Figure** **6**A). The azide‐MWCNTs formed a perfect surface for passivation with 1% casein which facilitated measurements in undiluted saliva samples. The possibility of tuning the conductivity and catalytic activity of graphitic carbon foils was exploited in DPV‐based sensing.^[^ ^143^ ^]^ It is known that the physicochemical properties of graphitic nanomaterials can be tuned by exfoliation and/or surface activation with specific functional groups. By using a strategy of partial exfoliation and surface activation with carboxyl groups, modified graphitic carbon foils have exhibited a high sensitivity to variations in the diffusion and kinetics of [Fe(CN)_6_]^3−/4−^ reaction (Figure 6B). This sensitivity was exploited for the detection of SARS‐CoV‐2 S proteins recognized by surface‐immobilized antibodies. The LOD was in the order of tens of pg mL^−1^ while the detection was conducted in artificial saliva.

In conclusion, Au nanostructures and doped or surface‐modified GO have extensively been used in voltammetric sensors. Lowering the detection limit of DPV and SWV sensors from the pg mL^−1^ level to a few fg mL^−1^ has been made possible by engineering nanostructures on Au surfaces or by forming heterostructures of two or more nanomaterials (see Table 2). On the other side, there was observed no gain in the detection range which shifted to lower protein concentrations. Especially for voltammetric sensors based on graphitic nanomaterials, the biocompatibility of nanomaterial surfaces to various methods of electrode passivation against non‐specific biomolecule binding, such as the surface modification with poly(ethylene glycol) or casein, has enabled the analysis of undiluted saliva samples. In summary, the analysis times with state‐of‐art voltammetric sensors are not inferior to 30 min and are mainly affected by the mass transport phenomenon of the analyte toward the electrode surface and by the use of a secondary biorecognition probe if a “sandwich‐type” immunoassay would be necessary for the target analyte.^[^ ^116^ ^]^

#### 2.4.1 Pros and Cons of Voltammetric Sensors

The pros of the voltammetric sensors for protein detection in saliva are 1) the wide availability of electrocatalytic nanomaterials for DPV and SWV electrodes, 2) the flexibility of assay formats either using single bio‐receptors or combining multiple bio‐receptors in one assay, thereby widening the possibility of targeting many types of proteins, 3) the readout of the voltammetric signals requires less processing compared to impedimetric sensors, and 4) the possibility of analyzing minimally processed saliva samples. The disadvantages of voltammetric sensors are related to 1) the dependence on the redox activity of an externally added probe and subsequent dependence on its diffusion and reaction kinetics, 2) the reduced charge transfer efficiency in the electrode caused by poly(ethylene glycol) or casein‐based surface passivation, and 3) the influence of orientation and polarity of target protein molecules on the charge transfer between the redox probe and the electrode.^[^ ^140^ ^]^

### 2.5 Electrolyte‐Gated Field‐Effect Transistor Sensors

EGTs are FETs in which an electrolyte acts as a gate insulator dielectric in contact with the conducting channel. In this setup, the ions in the electrolyte are displaced in opposite charges at the interface channel/gate when an electrical field is applied. In EGTs, nanomaterials are commonly applied to the FET‐conducting channel exploiting their superior charge transport characteristics.

**Figure 7:**
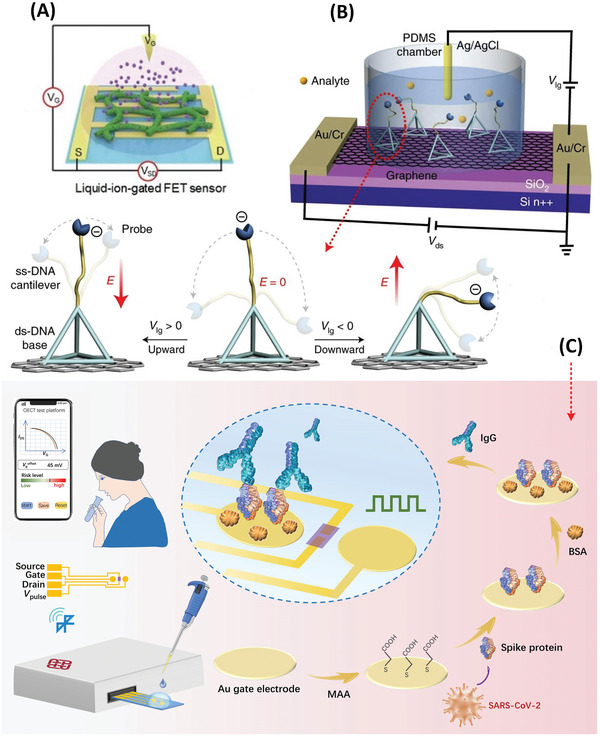
Electrolyte‐gated field effect transistors (EGTs). A) Detection of Nesfatin‐1 in saliva by an electrical double layer transistor (EDLT) comprised of multi‐pore carbon nanofibers deposited on interdigitated electrodes and functionalized by antibody. Reproduced with permission.^[^ ^121^ ^]^ Copyright 2021, Royal Society of Chemistry. B) A graphene‐based EDLT for protein detection with resolution in the attomolar level. In this sensor, the conformation of a flexible single‐stranded DNA (ssDNA) cantilever is adjusted according to ion‐gate voltage (*V*_lg_), bringing an aptamer probe bound to the analyte near the EDLT channel. Detection occurs within the Debye length. Reproduced with permission.^[^ ^146^ ^]^ Copyright 2022, Springer Nature. C) An organic electrochemical transistor for detection of salivary IgG with biorecognition occurring through the gate modified with antigen. Reproduced with permission.^[^ ^124^ ^]^ Copyright 2021.

The EDLT is the type of EGT in which the binding of the biorecognition element with the target protein causes variation of EGT‐channel charge density. Multi‐pore carbon nanofibers were synthesized for an EDLT conducting channel used for detecting nesfatin‐1, a biomarker of epilepsy.^[^ ^144^ ^]^ The sensor is shown in **Figure** **7**A. The carbon nanofibers were impermeable to ions, and the operation of the sensor was based on the formation of an ultra‐thin electrical double layer at the electrode/electrolyte interfaces. Artificial saliva was used as the electrolyte.^[^ ^121^ ^]^ At fixed gate voltage and fixed source‐drain voltage, the source‐drain current (*I*_sd_) decreased with the formation of protein (nesfatin‐1) and antibody (anti‐nesfatin‐1) immune complexes onto the surface of the carbon nanofibers. These immune complexes induced changes in the charge transport properties of the carbon nanofibers channel, decreasing the hopping rate of charges. The performance of this EDLT benefited from the porous structure of carbon nanofibers which increased the surface area for antibody binding, thereby detecting channel charge density variations with high sensitivity. Proteins were detected in the range of femtomolar (fM) concentration as the limit with this EDLT.

Graphitic nanomaterials are also specially designed for EDLTs to achieve conducting channels ultra‐sensitive to variations of charge in the vicinity of the channel. GO is one of the most reported graphitic nanomaterials for EDLTs. For the detection of human papillomavirus‐16 E7 protein in saliva, a GO EDLT channel was modified with an RNA aptamer.^[^ ^122^ ^]^ The change of conformation of the aptamer upon binding with the target protein enabled an increased *I*_sd_ response under a positive gate voltage. Before binding with the protein, the negatively charged RNA aptamer was folded in the vicinity of the FET channel, which counteracted the applied electric field leading to negligible alteration in *I*_sd_. The graphene monolayer is another nanomaterial with a high potential for ultra‐sensitive EDLTs. Figure 7B shows an EDLT with a graphene monolayer channel coupled to an advanced biomolecular structure for protein recognition (named DNA cantilevers). The DNA cantilever controls the induction of electric charges on the graphene monolayer which is known to be very sensitive to electrical potential variations.^[^ ^145^ ^]^ The target protein binds to an aptamer as part of the biological cantilever linked to a flexible single‐stranded DNA (ssDNA). The aptamer‐protein complex is detected under a negative potential across the electrolyte gate (*V*_lg_ < 0 in Figure 7B). At such potential, the negatively charged ssDNA moves downward bringing the aptamer‐protein complex within the Debye length in which the variation in the electrical potential of the graphene channel is maximum. By the measurement of *I*_sd_ which decreased with increasing protein concentrations before signal saturation, this EDLT detected thrombin in the range of 0.5 aM to 0.25 pM.^[^ ^146^ ^]^ In conclusion, the graphitic nanomaterial‐based EDLT exhibits ultra‐high sensitivity to protein detection in unprocessed saliva samples, on the condition that the biorecognition probe‐protein complexes are measured within the Debye length.

The ECT is another modality of EGT with the merit of ultra‐high sensitivity to salivary biomarkers´ detection within the Debye length. Organic semiconductors (OSCs) are a class of nanomaterials mostly employed as the ECT channel due to their high permeability to ions. The ECTs based on OSCs (or OECTs) are commonly characterized by their high transconductance, low working voltage, and excellent stability in contact with the electrolyte.^[^ ^98^ ^]^ When arranged as a FET channel the OSC withstands volumetric doping/dedoping upon injection of ions via the gate electrode. This unique feature makes the OECT ultra‐sensitive to minute binding events at the gate surface. With the immobilization of the target protein on the gate, large modulation of *I*_sd_ occurs due to the intrinsic charges of the protein or protein‐induced charge redistribution.^[^ ^147^ ^]^

Poly(3,4‐ethylenedioxythiophene)‐poly(styrenesulfonate) (PEDOT:PSS) is a hole‐conductive OSC widely utilized in OECTs. Upon application of a positive *V*_lg_, cations can be injected into the PEDOT:PSS channel, compensating for the depleted holes. A representative OECT with a PEDOT:PSS channel for salivary protein detection is depicted in Figure 7C. The sensor detected SARS‐CoV‐2 IgG on an Au gate electrode coated with S1 protein (hereby used as the biorecognition element). This OECT has exemplified the role of the intrinsic charges of the protein to induce a varied response of the OECT channel. The positively charged IgG bound to the S1 protein at the gate switched the *V*_lg_ to more negative potentials. The detection was demonstrated for eight orders of magnitude of IgG concentrations while the LOD was in the order of a few fM.^[^ ^124^ ^]^ As the IgG‐S1 protein complex has exceeded the Debye length in the unprocessed saliva sample, dilution was necessary to increase the Debye barrier.

Other conjugated polymers have been proposed to replace PEDOT:PSS in the OECT channel. A conductive polymer named p(g0T2‐g6T2) was introduced in an OECT for the detection of SARS‐CoV‐2 RBD.^[^ ^86^ ^]^ The new polymer allowed the ECT to be operated at even lower gate voltages than that in the PEDOT:PSS OECT, and with no cost in transconductance efficiency. Of remark, in addition to the new OECT channel, RBD was detected by nanobodies immobilized on the gate electrode. Due to their lower dimensions compared to antibodies, the nanobodies ensured the detection of the protein marker under the Debye barrier with no need for saliva sample dilution. The p(g0T2‐g6T2) OECT has achieved a single‐molecule sensitivity (in the range of zM) in unprocessed saliva samples. In conclusion, the OECTs provide a fast sample‐to‐result time (<15 min) in clinical samples with little or no sample processing, and their ultra‐high sensitivity is accompanied by the potential for device miniaturization.^[^ ^86^, ^124^ ^]^

Overall, nanomaterials are the essential components of the EGTs whose sensing performance demands the sensitive measurement of tiny changes in the FET channel conductivity. Among the graphitic materials reported for ion‐impermeable channel EGTs, the graphene monolayer may exhibit the highest potential. Coupling the graphene monolayer conducting channel with flexible single‐stranded oligonucleotide probes allows taking advantage of the ultra‐sensitivity of graphene to variations of charge in the vicinity of the material. For ion‐permeable EGTs, the progress in the synthesis of new conjugated polymers may deliver novel ion‐permeable materials enabling further improvement of detection limits and lowering the power to operate the FET. The operation of the EGTs concerning electrolyte gate voltages is generally dependent upon the charge mobilities of the conducting channel materials, the intrinsic charges of the target protein, and the ionic compositions of the electrolyte.

#### 2.5.1 Pros and Cons of Electrolyte‐Gated Field‐Effect Transistor Sensors

The major advantages of the EGTs for protein sensing in saliva are 1) the extremely high signal amplification in the conducting channel due to the ultra‐high conduction gain in graphitic nanomaterials (used in EDLT) and the volumetric coupling between ionic and electronic charges in the ECT channel, leading to ultra‐low detection limits, 2) the great biocompatibility of EGT nanomaterials, 3) the sensitive and accurate detection in unprocessed saliva samples taking advantage of sensing within the Debye length, 4) the high potential of the EGT to be coupled with integrated circuit designs, further miniaturizing the biosensor. EGTs also involve some disadvantages that need to be considered in the design stage of the biosensor, namely: 1) The dependence upon intrinsic charges of the target analytes which are defined by the respective isoelectric points in saliva. Any variation of protein charges between samples would significantly affect the reproducibility of detection; 2) less availability of nanomaterials compared to other electrochemical sensors, which limits design flexibility; 3) the possibility of *I*_sd_ signal saturation for protein concentrations exceeding the picomolar level.^[^ ^146^ ^]^

### 2.6 Photoelectrochemical Sensors

The PEC sensor is relatively new in the field of saliva‐based diagnostics.^[^ ^100^, ^148^ ^]^ Generally, PEC‐based protein detection involves the functionalization of a semiconductor electrode with bio‐receptors whose binding with the target protein induces variations of the sensor photocurrent response. Upon illumination, the semiconductor or semiconductor composite catalyzes the redox reaction of charge‐scavenging species at the electrode/electrolyte interface thereby generating the photocurrent response. TiO_2_, ZnO, and other wide bandgap semiconductors were commonly used in PEC biosensors because of their PEC stability. However, their low response in the visible light range has prompted a shift in the research toward semiconductors or semiconductor composites exhibiting a lower bandgap.

Graphitic carbon nitride (g‐C_3_N_4_) is a rising star nanomaterial in the field of PEC biosensing.^[^ ^149^ ^]^ It has a bandgap of 2.7 eV and allies great photosensitivity with excellent chemical stability. Due to its graphene‐like nature, g‐C_3_N_4_ possesses tunable electronic properties. Nevertheless, to achieve sufficiently high charge transfer efficiency, g‐C_3_N_4_ is normally associated with other semiconductors and metals forming PEC nanocomposites. A nanocomposite made of g‐C_3_N_4_, a perovskite (SrTiO_3_), and palladium (PD) nanoparticles were proposed for the sensitive detection of S1 protein in artificial saliva.^[^ ^125^ ^]^ The principle of detection involved the specific binding of S1 with an antibody coated on the composite surface which created steric hindrance to a redox probe (ascorbic acid) in solution. Upon illumination of the composite by visible light, and with no presence of S1 protein, photogenerated holes in the semiconductor composite were scavenged by ascorbic acid. In the presence of the S1 protein, the immune complex hindered the diffusion and kinetics of the redox probe at the PEC electrode/electrolyte interface, resulting in a decreased photocurrent response. The effect is proportional to increasing protein concentrations and it is an extensive strategy for any other protein marker. A major merit of this g‐C_3_N_4_‐based composite sensor was the achievement of a wide detection range covering fg mL^−1^ to ng mL^−1^ (six orders of magnitude).

Another composite of g‐C_3_N_4_ with CdS quantum dots was developed for the detection of RBD protein.^[^ ^127^ ^]^ The mechanism of interfacial charge transfer occurred similarly as in the aforementioned g‐C_3_N_4_/SrTiO_3_/PD sensor. The g‐C_3_N_4_/CdS composite surface was functionalized with a DNA aptamer and, in the presence of RBD, the bound biomolecular complex formed a barrier to the redox activity of ascorbic acid. One of the merits of the g‐C_3_N_4_/CdS PEC sensor was the good accuracy of detection as recovery rates exceeded 95% for saliva samples spiked with RBD.

**Figure 8:**
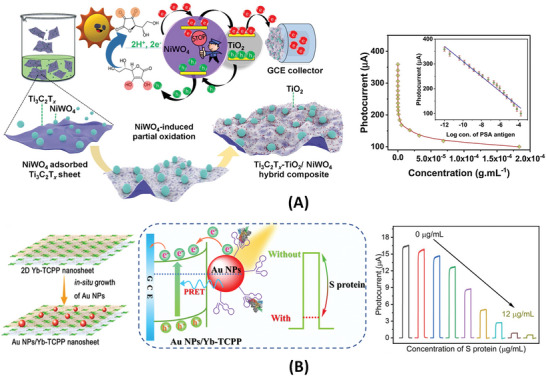
Photoelectrochemical sensors incorporating mixed‐dimensional nanomaterials. A) A photoelectrochemical sensor for prostate‐specific antigen targeted in saliva. The sensor is made of oxidized Ti_3_C_2_T*_x_* nanosheets with adsorbed NiWO_4_ nanoparticles. The heterostructure is used to modify the surface of a glassy carbon electrode‐GCE. Reproduced with permission.^[^ ^126^ ^]^ Copyright 2021, Elsevier. B) A photoelectrochemical sensor to detect S proteins based on the heterostructure of a 2D metal‐organic framework (Yb‐TCPP nanosheets) covered with AuNPs. Photocurrent response was enhanced via plasmon‐induced resonance energy transfer‐PRET. Reproduced with permission.^[^ ^151^ ^]^ Copyright 2021, American Chemistry Society.

Another graphite‐like nanomaterial with a 2D structure, namely Ti_3_C_2_T*_x_* nanosheets, was synthesized for a PEC sensor targeting the prostate‐specific antigen (PSA) cancer biomarker in saliva (**Figure** **8**A). For the electrode fabrication, Ti_3_C_2_T*_x_* was stabilized by partial oxidation in the presence of NiWO_4_ nanoparticles coating the nanosheets. Besides exhibiting high stability, the Ti_3_C_2_T*_x_*‐based PEC sensor has achieved protein detection covering fg mL^−1^ to sub‐mg mL^−1^ concentrations (eleven orders of magnitude) exploiting steric hindrance of PSA/antibody complexes on the redox activity of ascorbic acid. The ultra‐wide range of detection was attributed to the efficient charge transfer between Ti_3_C_2_T*_x_* nanosheets and the supporting GCE and between Ti_3_C_2_T*_x_* and the electrolyte and also due to reduced charge recombination in the oxidized Ti_3_C_2_T*_x_*. The LOD of this graphitic nanomaterial‐based sensor was below 1 fg mL^−1^.

Composites based on 2D metal–organic frameworks (MOFs) are another emerging class of materials for highly sensitive and stable PEC sensors.^[^ ^150^, ^151^ ^]^ In the nanosheet form, a 2D MOF named Yb‐TCPP was developed for PEC sensing of S1 proteins (Figure 8B). The Yb‐TCPP semiconductor nanosheets displayed high electron‐hole separation (quantum) efficiency and good electrode charge transfer properties, especially in combination with AuNPs. The Schottky junction formed by the 2D Yb‐TCPP and AuNPs enhanced the photoelectric conversion in the PEC electrode due to the phenomenon of plasmon‐induced resonance energy transfer (PRET). PRET is considered one of the state‐of‐art mechanisms for signal enhancement in PEC sensors.^[^ ^100^ ^]^ To maximize the enhancing effect of PRET, the 2D MOF was designed with an absorption spectrum overlapped with the surface plasmon resonance spectrum of AuNPs. A DNA aptamer was coated on the surface of AuNPs for the specific detection of S1 protein; the aptamer/protein complex formed a barrier to charge transfer between the composite electrode and a Na_2_SO_4_ electrolyte containing a redox probe. This work^[^ ^151^ ^]^ exemplified the amplification effect of AuNPs in PEC sensors not only for the photocurrent signal but also for the surface binding area.

In general, the analysis times for PEC sensors exceed 30–40 min and are majorly limited by the incubation times of the target proteins. To handle the saliva samples, the PEC working electrode is commonly passivated with a BSA solution after surface immobilization with biorecognition probes.^[^ ^125^, ^151^ ^]^ Although being pre‐treated with BSA, the majority of PEC sensors reviewed have not analyzed unprocessed samples. The PEC detection is still demonstrated for saliva in its diluted form or pre‐filtered by porous membranes.^[^ ^126^, ^127^ ^]^ The electrolyte solution containing the redox probe is commonly loaded on the PEC sensor after the incubation step with the analyte present in the saliva sample. Nevertheless, the good electrolytic properties of saliva biofluid^[^ ^80^ ^]^ open the possibility of adding the redox probe (i.e., ascorbic acid) into the saliva sample which reduces the number of incubation steps.

In conclusion, despite the intensive research on g‐C_3_N_4_‐based composites, Ti_3_C_2_T*_x_* as part of a new family of 2D metal carbides has shown the highest promise among the nanomaterials reported for PEC sensing of salivary proteins. The reason for the higher catalytic activity of this metal carbide compared to g‐C_3_N_4_ might be related to the exposed Ti sites on the metal carbide nanosheets which endow stronger redox reactivity than that of carbon‐based materials.^[^ ^152^ ^]^ Higher reactivity might justify the wide detection range achieved by Ti_3_C_2_T*_x_*. Nonetheless, due to the low cost of g‐C_3_N_4_, research on g‐C_3_N_4_‐based composites shall continue with the goal of realizing new PEC electrodes with improved detection range and sensitivity. Moreover, future works may exploit the use of saliva as the electrolyte medium and compare PEC sensing in saliva to that in standard electrolytes. It is also observed that other PEC sensing mechanisms, besides PRET and steric hindrance, namely in situ generation of electroactive or insulating compounds^[^ ^148^ ^]^ have not been much explored in saliva biosensing.

#### 2.6.1 Pros and Cons of Photoelectrochemical Sensors

The main pros of PEC sensors are 1) wide detection range for protein detection, 2) low LOD below fg mL^−1^ which can cover the physiological concentration limit of most protein markers in saliva, 3) fast signal generation from redox reactions at the electrode/electrolyte interface, 4) variety of sensor designs with multiple electrode architectures and suitability to various biorecognition elements. The major pitfalls are: 1) The complexity of PEC electrodes that is necessary to achieve the desired photocatalytic efficiency; 2) the PEC stability is affected by the pH and ionic strength of the electrolyte; 3) the addition of electroactive reagents for the sensing mechanism which limits automation of the assay; and 4) the need for processing the saliva sample before testing which increments assay steps and hinders rapid detection.

### 2.7 Comparison of Electrochemical Sensors for Salivary Protein Detection

The amperometric, potentiometric, impedimetric, and voltammetric sensors are classical electrochemical biosensors for salivary protein measurement. All of these sensors take advantage of signal enhancement with the incorporation of nanomaterials and most of them can perform satisfactorily in minimally processed saliva samples by implementing nanomaterial surface modification with blocking agents against non‐specific adsorption of interfering compounds (which may include large glycoproteins or other untargeted biomolecules naturally occurring in saliva). However, the amperometric and potentiometric sensors namely those based on enzymes for signal generation are more susceptible to variations in pH and temperature compared to the impedimetric or voltammetric analogs. On the other hand, in label‐free impedimetric and voltammetric utilizing steric hindrance as the sensing mechanism, the orientation and charge of the target protein molecules are relevant factors that can interfere with the detection signals. For these cases, there are practical solutions to minimize these interferences with the electric control of bio‐receptor surface immobilization^[^ ^153^ ^]^ and the standardization of background ionic composition of saliva with an associated cost in adding a step of sample pre‐treatment.^[^ ^154^ ^]^ With the minimization of interferences, the label‐free impedimetric and voltammetric sensor can perform more accurately and precisely than the enzyme‐based amperometric or potentiometric sensor.

The mode of detection and the automation of assay are also factors of differentiation among the above‐mentioned sensors. The continuous mode of detection for protein biomarkers in situ is needed in various clinical situations, including but not limited to patient safety against infections or organ failure by measuring inflammatory biomarkers.^[^ ^155^ ^]^ Potentiometric sensors and tethered amperometric sensors with near real‐time response and incorporating reversible binding bio‐receptors^[^ ^107^, ^156^ ^]^ have the perfect characteristics for analyte sensing in continuous mode. Furthermore, by analysis of existing assay protocols, the tethered sensor with an embedded redox reporter offers the best potential for assay simplification and subsequent automation even compared to recent label‐free impedimetric and voltammetric sensors that still require the addition of redox reagents.

Despite the merit of rapid responses of potentiometric and amperometric sensors, their limits of detection are seldom to be lower than the pg mL^−1^ level and their detection range is situated above 1 pg mL^−1^. There are cases of salivary biomarkers, for instance, interleukin‐6 or SARS‐CoV‐2 N protein, whose lowest concentration with clinical relevance was detected below pg mL^−1^.^[^ ^47^, ^67^ ^]^ Other biomarkers including but not limited to NT‐proBNP and TNF‐*α* have their lowest concentration close to 1 pg mL^−1^, and the variability in the biomarker determinations among subjects and between clinical studies needs to be considered. Therefore, achieving biosensor resolutions and detection ranges covering levels below pg mL^−1^ would ensure a “safe zone” for the analysis of salivary biomarkers. The detection of salivary proteins in the sub‐pg mL^−1^ and fg mL^−1^ concentration has been made possible with nanomaterial‐based impedimetric and voltammetric sensors (Table 2).

EGTs and PEC sensors are emerging classes of electrochemical sensors with a great prospect for continuous innovations in this field. To date, among the types of electrochemical sensors hereby reviewed, the EGTs may give the best compromise between low LODs, simplicity of assay formats, rapid detection, and amenability for testing unprocessed saliva samples. On the other side, the PEC sensor would find great applicability in clinical cases, in which is necessary to analyze biomarkers across a wide range of concentrations. The PEC sensor may potentially achieve accurate protein measurements from fg mL^−1^ to near mg mL^−1^.^[^ ^126^ ^]^ This feature relates to the high signal‐to‐noise ratio of the sensor due to the physical separation of light input from the electrical output. Determinations across a wide detection range with the same sensing principle can enable the analysis of multiple biomarkers or different disease conditions from one sensing platform.

## 3 Current Trends and Future Perspectives

In conformity with the recent research progresses in saliva‐based electrochemical sensors being reviewed in Section 2, the following discussion focuses on trends and key factors for the advancement of electrochemical biosensors based on nanomaterials. Improving the detection sensitivity and the detection limit, reducing the analysis time with minimal sample handling, and reaching the automation and miniaturization of the biosensor are common goals for researchers and engineers to achieve in clinically useful POC devices.

### 3.1 Modifying Electrodes with Nanomaterials

Nanostructured Au, carbon nanostructures, graphitic nanomaterials (GO, MWCNTs, g‐C_3_N_4_), and conjugated polymers are among the rising nanomaterials used for modifying Au and SPEs or MEMS interdigitated electrodes and thereby achieving the desired performance. Certain types of electrochemical sensors have been predominantly adopting a specific class of nanomaterial. For instance, graphitic nanomaterials are widely used in DPV sensors due to their easily tunable electronic properties and fast response to induced conductivity changes.^[^ ^95^, ^116^ ^]^ On the other side, conjugated polymers are the optimal choice for ECT sensors, which is justified by their record‐high transconductance and volumetric balance between ionic and electronic charges.^[^ ^44^, ^99^ ^]^ Nevertheless, although the single‐nanomaterial‐based sensor may perform satisfactorily, a substantial number of works indicated the need to modify the nanomaterial with doping elements^[^ ^115^ ^]^ and/or form composites with another one or more nanomaterials.^[^ ^148^ ^]^ Especially, the design of heterostructures or nanocomposites of two or more nanomaterials is crucial in impedimetric, voltammetric, and PEC sensors to maximize charge transfer efficiencies at the electrode/electrolyte interfaces.^[^ ^111^, ^120^, ^125^ ^]^

The role of electrode materials is not dissociated from the biorecognition probes. Nanobodies and aptamer/peptide probes with fold/unfold states are currently exploited to ensure ultra‐sensitive detection within the Debye length. This is particularly relevant for the test of saliva or other biofluids in which the Debye length may recede to less than 1 nm.^[^ ^87^, ^147^ ^]^ The Debye length in an electrochemical sensor defines how far the electrostatic effect from a protein‐receptor complex on the response of a nanomaterial electrode persists. The bio‐receptors are also associated with electroactive labels. By labeling a secondary receptor, polydopamine for instance can act as an effective electron donor in an electrochemical sensor to further enhance charge transfer kinetics at the electrode/electrolyte interface, or to sweep holes at the interface to further reduce undesired recombination in electrodes made of semiconductors.^[^ ^158^ ^]^ On the other hand, the primary receptor bound on the nanomaterial surface can also immobilize enzymes that catalyze reactions generating charge donors in situ.^[^ ^159^ ^]^ In this case, the specific binding of these receptors to target proteins ceases this electroactive species generation.

### 3.2 Incorporating Signal Amplification Techniques

Significant efforts have been made to endow the working electrodes with protein detection sensitivities and LODs at the femtomolar or even some at the zeptomolar (single‐molecule) levels.^[^ ^86^, ^157^ ^]^ To obtain these levels of sensitivity and LOD, strategies of signal amplification have been adopted in the design of the nanomaterial‐based electrodes. The enhancement of the electron and mass transfer processes at the electrode/electrolyte interfaces are the desired effects with the use of signal amplification methods in electrochemical sensors.

The charge transfer efficiencies are controlled by the composition, surface modification, and dimensionality of the electrode materials. Several materials such as carbon nanotubes and graphene oxide, for instance, improve the electrical connection between the reaction sites and the electrode. Element doping is a flexible strategy to tune electron energy band structures of the employed nanomaterials.^[^ ^160^ ^]^ Moreover, the formation of nanocomposites aims at reducing the pitfalls and maximizing the strengths of individual electrode materials.^[^ ^118^ ^]^ Surface modification with metal and metal oxide nanoparticles not only enhances the conductivity of the electrode but also increases the reaction sites. The rising focus on 2D layered materials, with thickness reduction from bulk to single‐layer, allows exploiting the quantum confinement effect to tune the electronic properties.^[^ ^145^, ^161^ ^]^ Moreover, at the single‐layer or few multi‐layer forms, the nanomaterial may exhibit surface defects or lattice vacancies which can further improve the reactivity and conductivity of the electrode.^[^ ^161^, ^162^ ^]^

Besides the exploitation of nanomaterials, the response of the electrode can be magnified by electroactive reagents in solution. One approach is to exploit mediator shuttles^[^ ^163^ ^]^ which enhance the kinetics of the redox reactions on the electrode surface, thereby lowering the electrochemical potential. Another strategy is the design of two or more redox couples acting in synergy to continuously generate charge species and increase charge accumulation on the electrode.^[^ ^130^ ^]^ Nevertheless, the use of extra electroactive agents in the electrolyte comes with the cost of increasing the complexity and duration of the assay.

In electrochemical analysis and sensing, the mass transfer efficiencies of reactants or coexisting ions have an impact on the electrochemical response. Enhancing mass transfer on the electrode surface would make the electrode more sensitive to variations of redox kinetics and signal intensity at the time of introducing the target protein. Nanoporous structures, for instance by patterning Au in form of nanowires^[^ ^119^ ^]^ or nano‐concave structures,^[^ ^133^ ^]^ can reduce the distance of the interaction between the redox probe and active sites on the electrode, leading to a gain in reaction efficiency. Furthermore, engineering 2D materials with surface atom defects can tune the surface wettability of the electrode affecting the mass transfer (adsorption/desorption) of the analytes.^[^ ^162^ ^]^

The nanoconfinement effect observed in nanochannels can also alter the mass transfer processes for electrochemical sensing. For protein detection in saliva, nanoporous membranes with two open ends^[^ ^120^ ^]^ were introduced to increase the diffusion rate of a redox probe on the supporting electrode. Subsequently, the binding of biomolecules onto the nanoporous membrane was effective in reducing access of the electrode to the redox probe, thus altering the signal with high sensitivity. Moreover, the technique of “confined thin liquid layer”^[^ ^164^ ^]^ involving for instance the confinement of the electrode/electrolyte system between two planes can significantly accelerate mass transfer via incrementing the collision frequency of electroactive species with the electrode surface.

### 3.3 Realizing Assays with One‐Step Detection

Decreasing the number of assay steps and subsequently decreasing incubation times is essential for rapid protein testing at the point of care. Numerous electrochemical sensors exploit single antibodies or single aptamers bound to target protein to create steric hindrance to the diffusion and redox activity of probe species on the electrode surface.^[^ ^83^, ^109^, ^114^, ^143^ ^]^ Although the analysis times of these sensors are majorly controlled by the step of sample incubation, their operation typically involves adding an electrolyte reagent containing the redox probe or charge scavenger. One‐step detection would perfectly be realized with the conjugation of the redox probe to the biorecognition element. Ferrocene was bound to a negatively charged double‐stranded DNA (dsDNA) which served as an electrode surface linker to a regular antibody.^[^ ^131^ ^]^ The variation of the applied potential to this sensor allowed the manipulation of the distance between the redox probe and electrode surface thereby regulating the electrochemical signal. Another electric‐field manipulated sensor was produced by developing ssDNA cantilevers bound to rigid dsDNA structures.^[^ ^146^ ^]^ In these cases, the one‐step detection involved no addition of reagents which promotes the autonomous operation of the biosensor devices. Foldable biomolecular systems independent from the applied electric field also hold potential in electrochemical sensors with one‐step and reagent‐less detection features. One of the extremities of DNA/RNA aptamer can be conjugated with a redox molecule (commonly methylene blue) while the other extremity is bound to the nanomaterial electrode surface. Folding/unfolding of the aptamer by recognition of the target protein can alter the distance of the redox molecule from the sensor surface, and this event occurs with minimal effect from the electrical potential.^[^ ^165^ ^]^ The same phenomenon can be exploited by the use of reversibly folded single‐domain protein receptors with chemical groups conjugated to a redox‐active label.^[^ ^166^ ^]^ Overall, the above‐discussed one‐step assays normally retain their performance merits in full biofluid samples.^[^ ^131^, ^165^ ^]^

### 3.4 Handling Full Saliva Sample

Reducing the handling of the saliva‐based sensor after sample collection is a vision in POC settings. Detection in the full biofluid sample accelerates the assay, facilitates sensor automation, and promotes the user‐friendliness of the device. Saliva contains a significant amount of large glycoproteins, mucus, and debris which can be removed by centrifugation. Filters are also used to remove assay‐interfering particles and large molecules following protocols with or without centrifugation.^[^ ^126^ ^]^ Nevertheless, the dilution of the sample is a widely practiced method for minimizing interfering substances; moreover, it introduces benefits for electrochemical sensing such as the regulation of Debye length.^[^ ^96^ ^]^ Apart from the fact that dilution brings an additional step to the assay, it decreases the concentration of biomarkers from their original levels. This is critical for the detection of proteins in saliva at very low concentrations. The modification of the nanomaterial electrode surface has therefore been investigated for realizing practical and effective devices.

Coating the sensor surface with negatively charged DNA sequences has been demonstrated to minimize the binding of untargeted biomolecules in saliva.^[^ ^112^, ^167^ ^]^ Moreover, a more universal procedure is the modification of the electrode surface with SAMs and especially BSA. Decreased electron transfer at the electrode/electrolyte interface can be encountered in electrodes coated with traditional anti‐fouling molecules.^[^ ^168^ ^]^ The electrical response and sensitivity of the sensor may largely be preserved in devices in which the anti‐fouling molecules form an interlaced composite with conductive nanomaterials on the electrode surface. A porous 3D composite can be formed by cross‐linking BSA with glutaraldehyde and interlacing it with Au nanowires.^[^ ^169^ ^]^ The amperometric response preserved nearly 90% of its original signal after one month of exposure to unprocessed biofluid.

Reversible functionalization of electrode surfaces with immunomagnetic particles (IMPs) is another strategy for handling the full saliva sample.^[^ ^82^, ^170^ ^]^ IMPs capture the target protein in the sample and subsequently bring it to the vicinity of the electrode surface by the application of a magnetic field. Electrochemical detection occurs with the aid of enzymes labeling a detection antibody. This approach permits the reuse of the electrode although the long‐term operation may likely be hindered by the unspecific adhesion of biomolecules. The sensor architecture may also aid the anti‐fouling behavior of the sensor regardless of the type of surface modification applied. In the case of PEC sensors, for instance, the utilization of photo‐cathodes for electrochemical signal transduction reduces the interference of reductive agents (i.e., dopamine, uric acid, etc.) that are oxidized in the opposite electrode (anode).^[^ ^171^ ^]^

### 3.5 Developing Sample‐in‐Answer‐out Systems

Microfluidics is desirable not only for providing controlled transport of biofluid and reagents over the electrochemical sensor, ensuring accuracy and reproducibility of the signal but also for realizing the automation of the assay. A range of microfluidic devices has been introduced during the last decades for saliva handling and downstream biomarker analysis. The early microsystems were derived from the need for miniaturization and automation of ELISA plate protocols. To this end, microarray well chips were realized by silicon photolithography or poly(dimethylsiloxane) (PDMS) soft lithography and coupled to flow cell apparatuses.^[^ ^28^, ^33^, ^172^ ^]^ Microporous agarose beads were immobilized on the microwells to enlarge surface areas and reduce assay times.^[^ ^28^, ^172^ ^]^ Other concepts include the lab‐on‐disc (L‐O‐Disc) and the passive‐flow polymer microchip (P‐F‐Chip). L‐O‐Disc employs centrifugal pumping force to transfer samples and reagents between multiple zones of the microfluidic chip intended for different steps of the assay.^[^ ^30^ ^]^ The L‐O‐Disc was essentially created to improve reaction kinetics in sandwich immunoassays. P‐F‐Chip is based on capillary flow assays with on‐chip dried reagents. A representative example of a P‐F‐Chip integrates microchannels for sample loading, capillary pumps for fluid waste removal, dried detection antibodies, time delay microchannels for controlling incubation times, and circular‐shaped reaction chambers coated with capture antibodies for the bio‐detection.^[^ ^173^ ^]^ The concept can reduce the time and improve the accuracy of sandwich‐type amperometric assays.^[^ ^174^ ^]^

Great focus is increasingly spent on the integration of the electrochemical sensor into paper‐based microfluidic platforms. Two approaches have been adopted in this regard: the first involves the attachment of SPEs with channels and reaction areas arranged on the paper substrate which can be regular filter paper or nitrocellulose strip;^[^ ^175^, ^176^ ^]^ the second deals with the impregnation of the paper substrate with electrode materials which can be performed by screen printing for electrodes in the millimeter dimension range or by inkjet printing for analytical strips in the micrometer range.^[^ ^177^, ^178^ ^]^ Laser scribing can also be exploited to realize electrodes made of carbon in the paper substrate.^[^ ^178^ ^]^ The second approach may offer unlimited prospects regarding the creation of rapid and disposable electrochemical sensors; it is associated with very low production costs and simplifies the integration of sensing and microfluidic parts. The capacity of the porous structure of the paper to incorporate both the conductive materials and preloaded reagents makes a perfect platform for one‐step operating sensors.^[^ ^179^ ^]^

Minimal user intervention in the analysis of saliva samples at the point of care motivates the realization of sample‐in‐answer‐out systems. For “sample‐in”, the microfluidic platform can be integrated into a higher fluidic structure in which a user‐friendly saliva aid collector can be inserted and the sample loaded.^[^ ^180^ ^]^ For “answer‐out”, a portable and miniaturized potentiostat can be idealized with the results wirelessly sent to a personal smartphone or tablet (Figure 7C).

### 3.6 Achieving Multiplexed Detection

The accurate and precise diagnosis of many infectious and chronic diseases may demand the analysis of multiple proteins simultaneously.^[^ ^26^, ^73^, ^76^, ^181^ ^]^ Optical transduction using either fluorescence or absorbance is predominantly exploited by researchers to realize multiplexed detection in saliva.^[^ ^33^, ^172^ ^]^ A concept of L‐O‐Disc coupled with a bead‐based sandwich immunoassay has simultaneously detected three cardiac biomarkers, CRP, cTnI, and NT‐proBNP from one sample of saliva.^[^ ^30^ ^]^ Carboxyl‐functionalized microspheres encoded with different combinations of fluorescent dyes have been effective in detecting ten salivary proteins.^[^ ^33^ ^]^ These fluorescence‐encoded microspheres were run in PDMS/glass microchips and performed a parallel analysis of low‐abundance proteins (namely interleukins) at the pg mL^−1^ range from saliva obtained from patients. Moreover, the concept of digital ELISA has recently emerged as a promising technique for analyzing multiple salivary proteins simultaneously and at femtomolar or below concentration levels.^[^ ^182^ ^]^ Combined with microfluidic flow cytometry, digital ELISA with dye‐encoded beads may detect hundreds of proteins from sample volumes of a few µL.

Multiplexed measurements of salivary proteins by electrochemical sensors have been reported although their throughput capacity has generally been inferior to that exhibited by current optical sensing platforms. Practically, many multiplex electrochemical assays are still confined to a series of diluted concentrations of the same protein biomarker rather than analyzing different biomarkers in one platform. Amperometric sensors arranged in arrays of Au electrodes have been sought as a solution;^[^ ^175^, ^183^ ^]^ however, these multiplexed amperometric sensors were confined to measurements of salivary IL‐8. Another amperometric sensing platform using dual SPEs functionalized with double‐walled carbon nanotubes has simultaneously detected IL‐1*β* and TNF‐*α*.^[^ ^184^ ^]^ Other electrochemical techniques have also been exploited for multiplexed electrochemical measurements. DPV was coupled with an eight‐sensor array made of carbon SPEs; nevertheless, its analytical demonstration was limited to the analysis of various concentrations of the same analyte (RBD protein).^[^ ^117^ ^]^ A multiplexed fluidic‐impedimetric sensor was formed by combining nanostructured Au electrodes and MIPs;^[^ ^185^ ^]^ hereby, the analytical performance was only shown for SARS‐CoV‐2 spike protein despite the promises of parallel biomarker analysis.

There is still room for advancing electrochemical sensors with high‐throughput capability for multiple salivary biomarkers. The recent progress of one‐step assays, the realization of nanocomposites with anti‐fouling properties, and the viability of low‐cost production of nanomaterial‐based electrodes^[^ ^186^, ^187^ ^]^ and integrated microfluidic platforms^[^ ^188^, ^189^ ^]^ may in synergy contribute to the further development of multiplex electrochemical sensing in complex biofluid samples. Apart from the engineering developments, continuous discovery of new salivary biomarkers will itself stimulate the emergence of novel multiplexed sensor technologies and in the meantime widen the screening and diagnostic scope of saliva. Followingly, rigorous population studies will be demanded not only to confirm the clinical usefulness of the biomarkers but also to validate the responses of the sensors in clinical and non‐clinical situations. In the future, multiplexed electrochemical detection in saliva may likely be in connection with medical big data and artificial intelligence to maximize the usage of biomarker data in the context of POC settings.

## 4 Conclusion

The recent SARS‐CoV‐2 pandemic, along with urges for stricter monitoring of various chronic diseases and other infections, have elucidated the need for progressing saliva diagnostics to clinicians and engineers. The detection of protein markers and antigens in saliva offers unlimited opportunities to conduct fast screening or fast diagnosis that can accelerate proper medications, allow more precise treatments, and enable more effective plans of disease monitoring or contingency on a large scale. It is acknowledged that the traditional protein assays based on ELISA, LC‐MS, or spectroscopy fail in providing the desired rapid response; meanwhile, the applicability of most saliva‐based biosensors is currently hindered by lengthy detections, suboptimal performance in the full biofluid sample, lack of automation, and lack of high throughput. The electrochemical sensor still holds promise to rise to these challenges. Recent advances in electrochemical sensing have been presenting unique sensor designs with desired characteristics of one‐step operation, anti‐fouling, assay time of few minutes, wide detection range, and zeptomolar‐level LOD. Nanomaterials or their related nanocomposites have played a crucial role in boosting the performance characteristics of the electrochemical sensor while allowing it to retain simplicity, low cost, and high scalability.

This review highlights and discusses the paramount achievements of saliva‐based electrochemical sensors based on protein biomarkers. Despite the efforts spent in modernizing all types of electrochemical sensors with enhanced sensitivity and specificity, there are still gaps to fill up for achieving truly sample‐in‐answer‐out systems in POC settings. Microfluidics and multiplexing methods have yet to be further explored along with the discovery and validation of new salivary biomarkers. New material composites with bio‐functionalization in conjugation with more autonomous devices and advanced data mining are expected to evolve in the next years, which will benefit the evolution of the electrochemical biosensor and the rapid growth of electrochemical sensing implementation in healthcare.

## Conflict of Interest

The authors declare no conflict of interest.
